# Deciphering the regulatory landscape of enhancer RNAs in health and disease

**DOI:** 10.1038/s41392-025-02436-z

**Published:** 2026-01-29

**Authors:** Qian Wang, Peter ten Dijke, Chuannan Fan

**Affiliations:** Oncode Institute and Department of Cell and Chemical Biology, Leiden University Medical Center, Leiden, the Netherlands

**Keywords:** Epigenetics, Non-coding RNAs

## Abstract

Enhancers are distal *cis*-regulatory elements that orchestrate spatiotemporal gene expression patterns in response to developmental cues and environmental stimuli. Genetic and epigenetic alterations in enhancers are associated with the initiation and progression of human diseases, including cancers. Over the past few decades, accumulating evidence has revealed that a class of nascent RNA transcripts, known as enhancer RNAs (eRNAs), is broadly transcribed from active enhancers. These eRNA species contribute to complex and dynamic gene regulatory networks under both physiological and pathological conditions through diverse mechanisms. Notably, dysregulated eRNA expression has been reported across various cancer types and is often correlated with patient survival outcomes. Consequently, eRNAs are emerging as promising biomarkers and therapeutic targets for cancer treatment. This review provides a comprehensive summary of the current understanding of eRNAs and their mechanisms of action in gene regulation. We discuss the critical roles of eRNAs in both health and disease and highlight their diagnostic and prognostic value, as well as their therapeutic potential in cancer. Additionally, we review current strategies for targeting RNA transcripts, including eRNAs, and discuss the major challenges in developing eRNA-targeted therapies. Finally, we propose future directions for advancing eRNA-based interventions in the treatment of human diseases, including cancer.

## Introduction

Gene transcription is a highly dynamic and tightly controlled process.^1,2^ Despite sharing an identical set of genetic material, cells establish diverse gene expression patterns in response to developmental, environmental, and intrinsic cellular signals.^2,3^ This process is primarily regulated through the precise binding of transcription factors (TFs) to specific DNA sequences, collectively known as transcriptional regulatory elements (TREs), which include promoters, enhancers, and silencers.^4–6^ Promoters are DNA sequences that serve as primary docking sites for RNA polymerase and TFs to initiate gene transcription.^2,7–10^ Typically, promoters are positioned upstream of the transcription start site (TSS) of their target genes and often contain conserved sequence motifs, such as the TATA box, initiator elements, and CpG islands.^9,11^ Unlike promoters, enhancers are often located distal to their target genes but regulate transcription by facilitating chromatin looping or recruiting cofactors to interact with promoters.^12,13^ The coordinated interplay between these elements ensures the precise spatiotemporal control of gene expression. Disruptions in this regulatory network can lead to aberrant gene expression, contributing to developmental disorders and diseases such as cancer.^14–16^

Advancements in high-throughput sequencing and powerful clustered regularly interspaced short palindromic repeats (CRISPR)-Cas-based gene and RNA editing techniques have made it feasible to gain deeper insights into the human genome, facilitating the discovery of a wide variety of novel RNA transcripts and a comprehensive characterization of their functions.^17–19^ Among these RNA species, enhancer RNAs (eRNAs), transcribed from active enhancer regions, play a critical role in activating target genes through modulating the functions of their corresponding enhancers.^20–23^ Recent large-scale transcriptome analyses have revealed the dynamic expression landscapes of eRNAs across various human cancers,^24–27^ underscoring their potential clinical utility in a cancer type-specific context. In this review, we provide a comprehensive overview of the history, biogenesis, and regulation of eRNAs and summarize the current methods employed for their detection and investigation. Additionally, we discuss the diverse functions and mechanisms of eRNAs in gene regulation, highlighting their critical role in both health and disease. In particular, we focus on their diagnostic and prognostic values, as well as their therapeutic potential in cancer. Finally, we review current strategies for targeting RNA transcripts, including eRNAs, and discuss the major challenges in developing eRNA-targeted therapies.

To obtain a broader perspective on eRNAs and RNA-based therapy, we refer our readers to several excellent reviews that focus on the discovery and progress in enhancer biology,^27–33^ approaches to comprehensively study eRNAs,^34^ and approaches that target non-coding RNAs for cancer therapy.^35–39^

## Enhancers and gene regulation

In response to distinct developmental and environmental signals, cells exploit unique DNA elements, including promoters, enhancers, and silencers, to control gene expression networks.^40–43^ Enhancers are defined as *cis*-regulatory DNA elements that can activate target gene expression over long distances.^34,44,45^ The first enhancer was cloned and functionally characterized from the simian virus 40 (SV40) genome in 1981.^46,47^ As estimated by the ENCODE Consortium, more than 400,000 enhancers exist in the human genome.^48–50^

Enhancer sequences are modestly conserved across species, making it challenging to predict their functional units on the basis of primary sequence analysis.^51–56^ The activity of enhancers is highly dynamic and confined to certain cell types and environmental stimuli.^45,57,58^ Active enhancers share several common features: high chromatin accessibility marked by DNase I hypersensitive sites^59–61^; a high ratio of the histone marker H3 lysine 4 monomethylation (H3K4me1) to H3 lysine 4 trimethylation (H3K4me3)^62,63^; high H3 acetylation at lysine 27 (H3K27ac)^64–66^; and the occupancy of transcription factors, transcriptional coactivators, including p300/CREB-binding protein (CBP) and bromodomain containing 4 (BRD4), and RNA polymerase II (RNA pol II).^67–72^ By establishing connections with corresponding promoters through three-dimensional (3D) physical chromatin interaction, enhancers promote the assembly of the transcriptional machinery complex at target promoters to facilitate transcriptional initiation.^30,73,74^ In addition, enhancer–promoter looping contributes to transcription elongation by regulating the pause release of RNA pol II.^12,75,76^

## eRNAs act as another layer of enhancer function

### The discovery of eRNAs

**Fig. 1:**
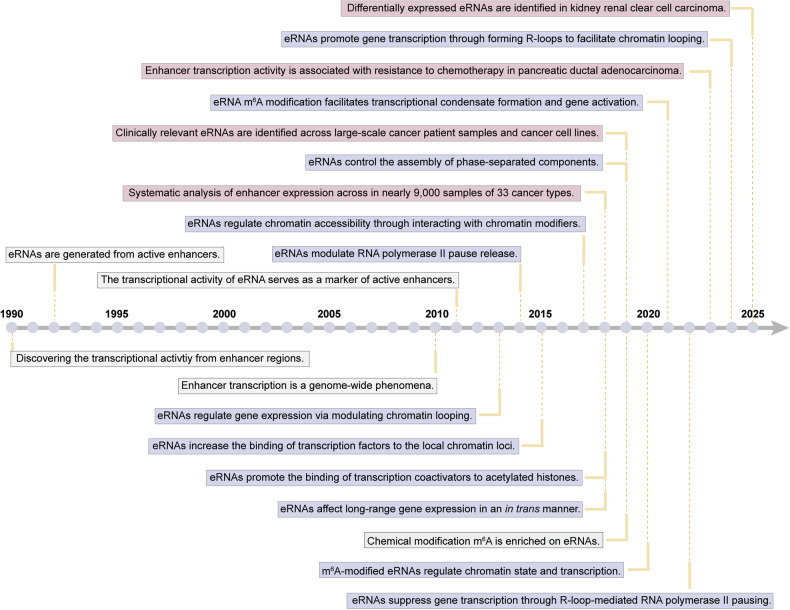
Milestones in the path toward discovery and functional characterization of eRNAs. This figure highlights key advancements in the field of eRNAs from 1990–2025. Major biological discoveries, including the identification of eRNAs and their modifications, are represented in gray boxes. Breakthroughs in understanding the mechanisms of eRNA-mediated gene regulation are depicted in purple boxes. The clinical significance of eRNAs is indicated in pink boxes

Studies in the early 1990s revealed transcriptional activity at enhancer regions^77^ and discovered that active enhancers can give rise to non-coding transcripts to maintain their activities.^78^ However, the global recruitment of RNA pol II to enhancers and the genome-wide transcription of eRNAs from active enhancers were reported from 2010^79,80^ and onward^81–83^ (Fig. 1). Genome-wide sequencing methods have revealed that membrane depolarization stimulates the activation of neural activity-regulated enhancers marked with H3K4me1 and CBP enrichment in mouse cortical neurons.^80^ The neural enhancers can transcribe bidirectional eRNAs, whose expression is positively correlated with that of messenger RNAs (mRNAs) encoded by their neighboring genes, indicating that eRNA synthesis serves as a marker of enhancer activation^80^ (Fig. 1). Another study revealed that inflammatory stimulation triggers the occupancy of extragenic RNA pol II to enhancers adjacent to inflammatory genes, producing low-abundance eRNAs from these inflammatory-related enhancers in mouse macrophages^79^ (Fig. 1). Afterwards, a growing number of studies revealed the stimuli-induced synthesis of eRNAs and their contributions to gene regulation across multiple cell types^58,84–87^ (Fig. 1).

### Classification of eRNAs

**Fig. 2:**
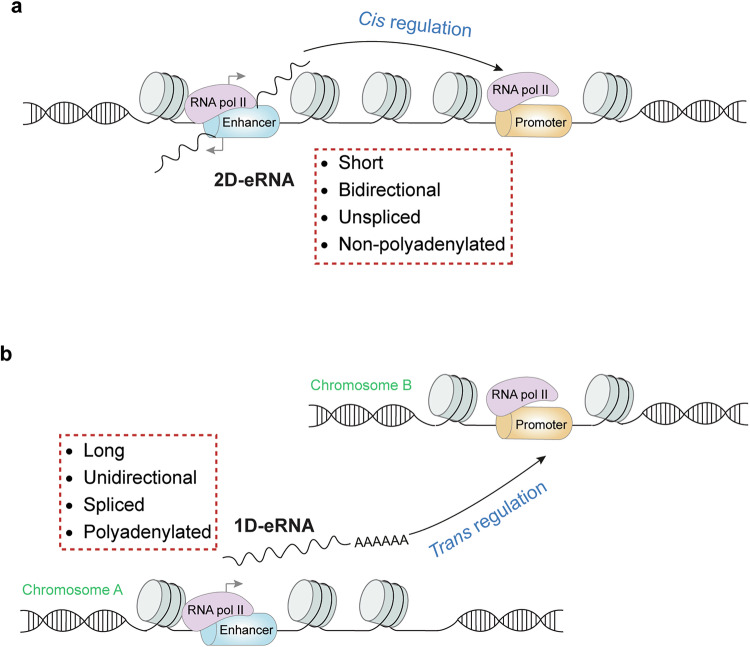
Molecular features of eRNAs. Schematic diagram showing the major differences between 2D-eRNAs (**a**) and 1D-eRNAs (**b**). RNA pol II RNA polymerase II

eRNAs are broadly classified into two subgroups with diverse characteristics. One subgroup, known as bidirectionally transcribed eRNAs or 2D-eRNAs, is typically short—usually less than 150 nucleotides (nt) in length—as determined by transcription termination assays.^22,88,89^ These eRNAs are nonspliced and lack polyadenylation.^22,88,89^ In contrast, the second subgroup, referred to as unidirectionally transcribed eRNAs or 1D-eRNAs, is considerably longer, ranging from 500 nt to over 2,000 nt.^79,85^ These transcripts are spliced and polyadenylated.^79,85,90^ Moreover, in most cases, 1D-eRNAs function in trans, whereas 2D-eRNAs act *in cis* (as discussed in the following sections)^22^ (Fig. 2). Owing to their weak transcription and high turnover rate, the cellular levels of eRNAs are lower than those of mRNAs and long non-coding RNAs (lncRNAs).^91,92^ Table 1 compares the key molecular features of mRNAs, lncRNAs, and eRNAs. eRNAs are transcribed from enhancers with low H3K4me3, but lncRNA transcription is driven by promoters with high H3K4me3 modification.^21,93,94^ Given the positive correlation between H3K4me3 and gene transcription,^95–97^ the difference in H3K4me3 levels between eRNA-derived enhancers and lncRNA promoters may explain the abovementioned lower expression of eRNAs than that of lncRNAs.^98^ Owing to the existence of longer eRNAs (>150 nt),^88,89^ the definition of eRNAs is not exclusive to lncRNAs, whose lengths are arbitrarily defined to be longer than 200 nt.^99^ In addition, experimental evidence has shown that eRNAs and enhancer-associated lncRNAs regulate target gene expression in a similar manner.^22,100,101^ Conversely, super-enhancer-derived eRNAs can function as lncRNAs,^102^ reinforcing the notion that eRNAs and lncRNAs are not mutually exclusive non-coding transcripts regarding their functions. Therefore, the following sections do not distinguish between eRNAs and enhancer-derived lncRNAs.

**Table 1:** Common features among mRNA, lncRNA and eRNA

Features	mRNA	lncRNA	eRNA
Splicing	Yes	Common	Rare
Polyadenylation	Mostly	Some	Some
Stability	High	Medium	Low
Conservation	High	Low to medium	Low
Tissue specificity	Low	High	Very high
H3K4me1	Low	Medium	High
H3K4me3	High	Medium	Low
H3K27ac	High	High	High
RNA polymerase II	Yes	Yes	Yes
DNase I hypersensitive site	Yes	Yes	Yes
Subcellular localization	Mostly cytoplasmic	Nuclear and cytoplasmic	Nuclear
Size	500–5000 nucleotides (nt)	200–10,000 nt	50–200 nt (1D); 500–2000 nt (2D)

## Biogenesis and regulation of eRNAs

### Transcription of eRNAs

**Fig. 3:**
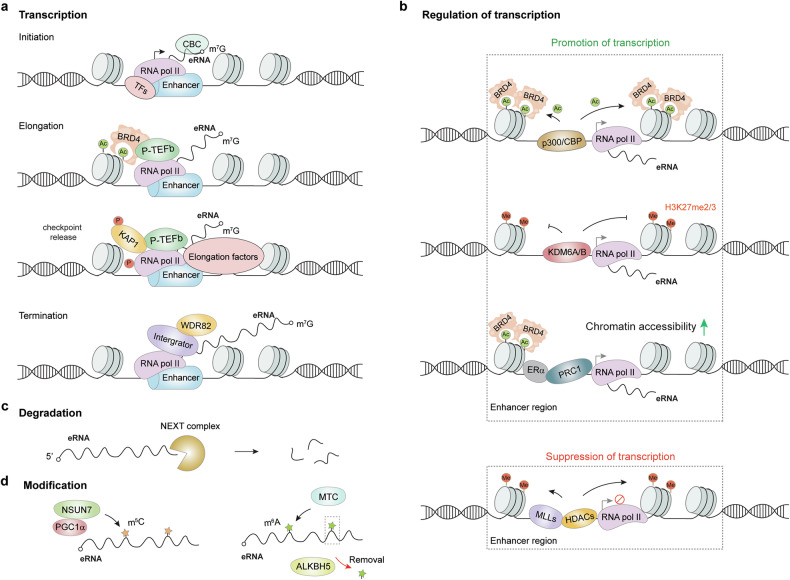
Biogenesis and modifications of eRNAs. **a** The transcription of eRNAs is initiated by the recruitment of TFs and RNA pol II at enhancer loci, after which the CBC binds to the 5’-end of eRNAs to assemble an m^7^G cap; BRD4 regulates the elongation of eRNA transcripts in a P-TEFb-dependent manner; the Integrator complex and WDR82 mediate the termination of eRNA transcription; **b** The regulatory network of eRNA transcription. The transcriptional coactivators p300/CBP, KDM6A/B, and PRC1 promote gene transcription, whereas MLLs and HDACs suppress gene expression. **c** The degradation of eRNAs is regulated by nuclear RNA exosome complexes. **d** Common chemical modifications of eRNAs. m^5^C modification of eRNAs is catalyzed by the PGCα/NSUN7 complex, and eRNA m^6^A is methylated by MTC and demethylated by ALKBH5. TF transcription factor, RNA pol II RNA polymerase II, CBC cap-binding complex, m^7^G 7-methylguanosine, P-TEFb positive transcription elongation factor, KAP1 Kruppel-associated box (KRAB)-associated protein 1, BRD4 bromodomain containing 4, WDR82 WD repeat domain 82, NEXT nuclear exosome-targeting complex, CBP CREB-binding protein, KDM6A/B lysine demethylase 6A/B, H3K27me2/3 histone H3 lysine di/tri-methylation, HDAC histone deacetylase, MLL mixed-lineage leukemia, NSUN7 NOP2/Sun RNA methyltransferase 7, PGC1α peroxisome proliferator-activated receptor-γ coactivator-1α, m^5^C 5-methylcytosine, m^6^A *N*^6^-methyladenosine, MTC methyltransferase complex, ALKBH5 AlkB Homolog 5, ac acetylation, me methylation, PRC1 polycomb repressive complex 1, ERα estrogen receptor α

eRNAs are transcribed in a manner highly dependent on cell type and environmental stimuli.^45,57,58,63,85,103^ eRNA transcription is initiated upon binding of certain transcription factors to accessible DNA elements at enhancer loci.^84,103,104^ These transcription factors then recruit transcription cofactors and RNA pol II to form a transcriptional complex to initiate transcription, after which the cap-binding complex (CBC) binds to the 5’ end of eRNAs to assemble a 7-methylguanosine (m^7^G) cap^105,106^ (Fig. 3a). In some cases, nucleosome remodeling occurs at nonactive enhancers to loosen DNA from nucleosomes and enable the binding of transcription factors to enhancers.^104,107,108^

eRNA elongation is generally mediated by mechanisms similar to those of protein-coding genes.^104,109,110^ In hyperacetylated enhancer regions, BRD4 interacts with the RNA pol II complex in a positive transcription elongation factor (P-TEFb)-independent manner to facilitate eRNA elongation^109,111^ (Fig. 3a). A recent study revealed a signal-dependent and ligand-dependent mechanism of eRNA elongation that involves the release of a conserved eRNA transcription checkpoint.^112^ In this process, the DNA-dependent kinase catalytic subunit (DNA-PKcs) phosphorylates Kruppel-associated box (KRAB)-associated protein 1 (KAP1), preventing its interaction with 7SK small nuclear ribonucleoproteins (snRNPs) and the SUMOylation of cyclin dependen kinase (CDK)9, the catalytic subunit of positive transcription elongation factor (P-TEFb).^112^ This activation of the P-TEFb complex facilitates the recruitment of elongation factors (Fig. 3a). This mechanism is observed when signal- or ligand-regulated eRNA transcription termination is tightly controlled by the cleavage of eRNAs from RNA pol II. The adaptor protein WD repeat domain 82 (WDR82) interacts with the cleavage and polyadenylation factor (CPF) to release synthesized eRNAs from RNA pol II^89,113^ (Fig. 3a). In addition, the multi-subunit Integrator complex interacts with the C-terminal domain of RNA pol II to cleave eRNA primary transcripts at their 3’ end^85^ (Fig. 3a). Notably, Integrator subunit 11 (INTS11), the catalytic subunit of the Integrator complex with endonuclease activity, plays a critical role in this process by cleaving nascent eRNA transcripts genome wide.^114^ Depletion of either WDR82 or Integrator leads to the accumulation of aberrant unprocessed eRNA transcripts, suggesting their contributions to eRNA maturation by controlling the transcription termination process.^85,89^

Epigenetic chromatin modifications widely regulate eRNA transcription. The transcriptional coactivator p300/CBP acetyltransferase occupies enhancer regions to alter the local histone acetylation landscape to trigger eRNA transcription^115,116^ (Fig. 3b). Histone acetylation is recognized by the histone acetylation reader BRD4, which promotes the release of paused RNA pol II into productive elongation at enhancers^109,117^ (Fig. 3b). Other coactivators, such as lysine demethylase 6A/B (KDM6A/B), also stimulate eRNA transcription by erasing histone H3 lysine di-/trimethylation (H3K27me2/3)^118^ (Fig. 3b). In breast cancer cells, the epigenetic regulator polycomb repressive complex 1 (PRC1) binds to oncogenic enhancer regions enriched with estrogen receptor α (ERα) or BRD4 and thereby promotes chromatin accessibility and facilitates eRNA expression^119,120^ (Fig. 3b). Consistently, PRC1 has been shown to localize to active enhancers that form 3D genomic loops with their target promoters in Drosophila and mouse models, highlighting a conserved role for PRC1–enhancer interactions in gene activation across species.^121^ DNA hypomethylation at enhancer loci has also been reported to facilitate eRNA transcription.^122,123^ Targeted demethylation of the *CCAAT enhancer binding protein-β* (*C/EBPβ*) enhancer promotes *C/EBPβ* eRNA expression in liver cancer cells.^122,124^ In contrast, eRNA transcription is suppressed upon the recruitment of the histone methyltransferases mixed-lineage leukemia (MLL)^104^ and histone deacetylases (HDACs)^58^ to enhancers (Fig. 3b).

### Degradation of eRNAs

The nuclear exosome-targeting complex (NEXT) can degrade eRNAs at the 3’ end, which may lead to a lack of polyadenine (poly(A)) signals (PASs) at the 3’ end of bidirectional 2D-eRNAs^91,92,125,126^ (Fig. 3c). Moreover, RNA exosome sensitivity negatively correlates with the distance between the TSS and the PAS.^127^ Hence, proximity between the TSS and PAS at enhancer loci may hinder the assembly of the polyadenylation machinery, resulting in subsequent rapid degradation of shorter 2D-eRNAs.^92,128^ This finding may explain why 1D-eRNAs with longer lengths are expressed at higher levels than shorter 2D-eRNAs.^91^

### Modification of eRNAs

Like other RNA species, eRNAs undergo various chemical modifications.^129,130^ A complex formed by peroxisome proliferator-activated receptor-γ coactivator (PGC)-1α and NOP2/Sun RNA methyltransferase 7 (NSUN7) facilitates the 5-methylcytosine (m^5^C) modification of PGC-1α-induced eRNAs in liver cells^131^ (Fig. 3d). m^5^C-modified eRNAs are stabilized and promote PGC-1α-induced transcriptional responses.^131^ As the most abundant internal RNA chemical modification, *N*^6^-methyladenosine (m^6^A) occurs on both coding and non-coding transcripts and plays a critical role in gene regulation.^132,133^ A whole m^6^A methylome study across 21 fetal tissues showed that over half of the eRNAs are enriched in m^6^A–seq data in most tissue types.^134^ Another study revealed that approximately 30% of eRNAs are marked with m^6^A in pancreatic ductal adenocarcinoma (PDAC) tissues.^135^ Moreover, methylation-inscribed nascent transcript sequencing (MINT–seq) revealed that m^6^A deposition is widespread but also selective on nascent eRNAs in multiple cell lines.^136^ The METTL3–METTL14–WTAP m^6^A methyltransferase complex (MTC) binds active enhancer regions to catalyze m^6^A methylation on eRNAs co-transcriptionally and thereby suppresses transcriptional termination to facilitate eRNA production.^137,138^ A recent study demonstrated that p300 acetylates METTL3 at H3K27ac–marked chromatin to prevent its binding to METTL14, thereby spatially inhibiting the m^6^A deposition of eRNAs.^139^ The m^6^A-eRNAs are demethylated by the m^6^A demethylase AlkB Homolog 5 (ALKBH5), which is enriched at enhancers with high MTC abundance, suggesting that the m^6^A modification of eRNAs is dynamically regulated^137^ (Fig. 3d).

### Overview of methods for eRNA identification and investigation

#### Methods for detecting eRNAs

The first evidence indicating the existence of eRNAs came from identifying a non-coding RNA transcribed from the stably integrated erythroid-specific *hypersensitivity site 2* (*HS2*) enhancer in K562 cells, using an RNA protection assay.^140^ This groundbreaking discovery laid the foundation for understanding transcription events at enhancers. Shortly thereafter, RNA fluorescence in situ hybridization (FISH) was employed to detect enhancer activity through direct visualization of RNA transcripts in fixed cells.^141^ Since then, FISH has become a cornerstone technique for elucidating RNA subcellular localization.^142,143^

Total RNA sequencing (RNA–Seq) measures the entire spectrum of cellular RNA species by high-throughput sequencing, whereas RNA–Seq with poly(A) enrichment (polyA^+^ RNA–Seq) selectively focuses on RNA species with poly(A) tails.^144,145^ Both methods are well-established techniques for detecting steady-state RNAs but are limited in capturing newly synthesized nascent RNA transcripts. PolyA^+^ RNA-seq filters out RNAs without poly(A) tails during the initial complementary DNA (cDNA) generation and library preparation.^144,145^ As a result, specific RNAs, for example, 2D-eRNAs,^79,90^ fail to be detected owing to their absence of polyadenylation. In contrast, analysis of chromatin-bound RNA, which avoids reliance on poly(A) selection, offers an effective strategy for enriching nonpolyadenylated, unstable, and short-lived transcripts such as 2D-eRNAs.^146^ Therefore, careful selection of appropriate techniques for eRNA detection is crucial to ensure accurate and comprehensive analysis.

Cap analysis of gene expression (CAGE) followed by deep sequencing is broadly used to define the initiation sites of eRNA transcription.^45,80,147,148^ Similarly, TSS sequencing combined with paired-end analysis of TSSs (PEAT) also allows the identification of the 5’ end of transcripts.^149^ However, these methods often require large quantities of high-quality RNA samples and may lack the sufficient sensitivity and accuracy needed to detect lowly expressed eRNAs.^149^ To address these issues, innovative approaches, such as global run-on sequencing (GRO-seq) and precision nuclear run-on and sequencing (PRO-seq), have been developed to capture nascent RNAs by mapping RNA polymerases that are actively engaged in transcription across the genome.^150–152^ The former can only map transcripts to genomic regions with moderate resolution, whereas the latter enables the pinpointing of exact RNA polymerase positions at single-nucleotide resolution. Precision Run-On and Capping (PRO-cap), a variant of PRO-seq, captures TSSs at the level of nascent RNA synthesis with high resolution, enabling more precise detection of unstable transcripts, including eRNAs.^153^ Small-capped RNA–seq, also referred to as START–seq, selectively detects 5’-capped nascent transcripts derived from stalled RNA polymerase, providing deeper insight into transcriptional initiation events.^154,155^ Complementing these methods, Native Elongating Transcript sequencing (NET-seq) permits the capture of the 3’ ends of nascent RNA attached to actively elongating RNA polymerase, which provides information on transcriptional dynamics across the entire gene, including elongation, pausing, and termination.^156,157^ Transient transcriptome sequencing (TT–Seq) is a powerful technique for detecting newly synthesized transcripts through metabolic labeling of nascent RNA, providing comprehensive insight into genome-wide transcriptional dynamics.^158^

#### Strategies for investigating eRNA functions

Various experimental approaches can be used to examine the functions of eRNAs. This section covers the commonly used methods for gain-of-function and loss-of-function studies as well as techniques for identifying the DNA and protein partners of non-coding RNAs, including eRNAs.

##### Gain-of-function studies

Ectopic expression of eRNAs offers possibilities for exploring their functions in trans.^159–162^ However, this approach often fails to mimic the role of *cis*-regulatory eRNAs, which act locally to influence the transcriptional activity of nearby target genes.^163^ To address this limitation, the CRISPR activation (CRISPRa) system can selectively upregulate the expression of desired eRNAs in situ through recruiting transcriptional activators to the targeted regions.^164,165^ However, this approach is confounded by the simultaneous activation of the enhancers themselves, making it difficult to distinguish direct effects from eRNAs and their corresponding enhancers. The CRISPR–Display system was developed to express eRNAs at targeted genomic loci.^166,167^ This system utilizes a nuclease-deficient mutant dead Cas9 (dCas9), which directs the eRNA of interest to the targeted genomic locus by coupling its sequence to the short guide RNA (sgRNA) sequence for its *in cis* expression.^167^ As expected, the CRISPR–Display system has demonstrated the activator effects of two eRNAs on genomic reporter activity.^166^ Combining the CRISPR–Display system with fluorescent RNAs (FRs) permits simple and robust imaging of the associated genomic loci as well as tracking real-time protein–RNA tethering in live cells.^168^ However, this approach is limited by the recruitment of the large dCas9 protein to enhancer regions, which may disrupt native interactions between eRNAs and their associated protein partners.

##### Loss-of-function studies

The expression of eRNAs can be suppressed by transcription elongation inhibitors such as flavopiridol^169^ and actinomycin D^170^ or by targeting BRD4 with bromodomain and extra-terminal domain inhibitors (BETis),^171^ such as JQ1,^172^ PFI-1^173^ and I-BET.^174^ However, these small-molecule compounds may affect the expression of protein-coding genes beyond eRNA expression per se, making it challenging to precisely determine the specific functions of eRNAs. In contrast, direct degradation of eRNA transcripts represents a more targeted and precise approach for elucidating their functions.^175–177^ This can be achieved through RNA interference (RNAi),^178^ RNase H-mediated knockdown with antisense oligonucleotides (ASOs),^179^ or the type VI CRISPR–Cas13 system.^17,19^ While RNAi-based approaches, such as short hairpin RNAs (shRNAs) and short interfering RNAs (siRNAs), are effective for targeting cytoplasmic RNA transcripts,^180^ ASOs and CRISPR–Cas13d are more suitable for degrading eRNAs, which are predominantly localized in the nucleus. Given that a significant proportion of eRNAs are marked with m^6^A modifications and that the loss of m^6^A marks can destabilize eRNA transcripts and influence their functional interactions,^135,181–183^ an m^6^A ‘eraser’ system has been developed to degrade eRNAs.^136^ This system fuses the m^6^A demethylase FTO (which removes m^6^A marks) to a catalytically inactive Cas13d (dCas13d).^136^ Additionally, manipulating eRNA-producing genomic regions by deleting large fragments or inserting a poly(A) cassette may also effectively disrupt eRNA transcription.^184–187^ Nevertheless, both methods may introduce confounding effects by disrupting the underlying enhancer sequence. Since the active transcription of eRNAs is closely linked to the presence of specific histone modifications at enhancer regions,^137^ the expression of specific eRNAs can be suppressed by the CRISPR interference (CRISPRi) strategy, which recruits transcriptional repressors or suppressive histone modifiers to the targeted genomic loci.^188–191^ However, these regulatory effects may also unintentionally impact the transcriptional activity of nearby genes or other non-targeted genomic regions.^192–195^

We summarize the advantages and limitations of each method used for eRNA functional characterization in Table 2. On the basis of this comparison, we propose that loss-of-function analyses using ASOs and CRISPR/Cas13d serve as primary strategies for the functional characterization of eRNAs. Complementary approaches, such as CRISPRa/i and CRISPR-Display, can provide additional support and validation for the findings obtained from ASO and CRISPR/Cas13d experiments.

**Table 2:** Overview of methods for eRNA identification

Methods	Purpose	Comments	Refs.
RNA fluorescence in situ hybridization (RNA-FISH)	Detect the transcriptional activity of enhancers	Single-cell-based method for visualizing RNA transcripts in fixed cells, but with low throughput and laborious	^141^
Total RNA sequencing (RNA-seq)	Measure the entire spectrum of cellular RNA species	High-throughput and well-established technique to detect the steady-state RNAs, but not robust at detecting the dynamic and transient transcriptional activity	^144,145^
RNA sequencing with poly(A) enrichment (Poly(A)^+^ RNA-seq)	Measure the RNA species with polyadenylated tails	Similar to RNA-seq, but fails to detect RNAs lacking poly(A) tails	^144,145^
Cap analysis of gene expression (CAGE) followed by deep sequencing	Define the initiation sites of eRNA transcription	Identify the exact position of eRNA transcription initiation using a small amount of material, but requires a large sample size for lowly expressed eRNAs	^45,80,147,148^
Transcription start site (TSS)-seq followed by paired-end analysis of TSSs (PEAT)	Identify the 5’ end of eRNA transcripts	Map the transcription sites with high resolution and comprehensive coverage, but not sensitive for detecting lowly expressed transcripts, and requires high sequencing coverage	^149^
Global run-on sequencing (GRO-seq)	Capture nascent RNAs	Detect unstable nascent transcripts with limited base resolution, and requires large amounts of material	^150^
5’ GRO-seq or GRO-cap	Capture nascent RNAs	Similar to GRO-seq, but provides precise start sites of transcription events	^150^
Precision nuclear run-on and sequencing (PRO-seq) and Precision Run-On 5′ Cap sequencing (PRO-cap)	Capture nascent RNAs	Similar to GRO-seq, but with relatively higher base resolution	^151,152^
START-seq	Detect 5’-capped nascent transcripts	Genome-wide coverage with high resolution, but may not capture uncapped RNA transcripts	^154,155^
TT-seq	Measure transient RNAs	Genome-wide view of nascent transcription with high temporal resolution, but it requires metabolic labeling and can introduce artifacts	^158^
Native elongating transcript sequencing (NET-seq)	Capture 3’ ends of nascent RNAs	Detect unstable transcripts and 3’ ends of RNA pol II-bound eRNAs at nucleotide resolution, but it is difficult to pinpoint the enhancer region by only detecting the 3’ end of eRNAs	^156,157^
CRISPR-Display	Tether eRNA to the predefined genomic locus	Ideal for testing the functions of eRNAs with multiple transcripts, but the gRNA scaffold has a limited capacity for incorporating additional RNA sequences	^166,167^
CRISPR activation (CRISPRa)	Activate endogenous eRNA expression	Selectively upregulate the expression of desired eRNAs in situ, but it may affect the local enhancer activity	^164,166^
Transcription elongation inhibitors	Suppress eRNA expression	Small molecule-based approaches may affect multiple pathways beyond eRNA per se	
Bromodomain and Extra-Terminal domain inhibitors (BETi)	Suppress eRNA expression		^169–174^
RNAi	Degrade eRNA transcripts	Selectively and efficiently degrade RNAs, especially cytoplasmic RNAs, but it may induce unintended intracellular changes	^178^
Antisense oligonucleotides (ASOs)	Degrade eRNA transcripts	Selectively and efficiently degrade cytoplasmic and nuclear RNAs, and chemical-modified ASOs are more stable	^179^
CRISPR/Cas13	Degrade eRNA transcripts	Selectively degrades RNA transcripts, but it may also affect the expression of other transcripts	^17,19^
CRISPR/dCas13-FTO	Remove m^6^A modification on eRNAs	Loss of m^6^A marks may destabilize eRNA transcripts	^136^
CRISPR/Cas9-based approaches	Suppress eRNA expression	Manipulate the eRNA-producing genomic regions by large fragment deletion or inserting a poly(A) cassette, but the introduction of double-stranded breaks (DSBs) via CRISPR/Cas9-based genome editing tools may trigger DNA damage and repair pathways	^184–187^
CRISPR inhibition (CRISPRi)	Suppress eRNA expression	Selectively downregulate the expression of desired eRNAs in situ, but it may affect the expression of neighboring genes	^188–191^
Chromatin-associated RNA (ChAR)-seq	Map RNA-chromatin interactions	Provide a comprehensive view of RNA localization relative to chromatin, but the experimental procedure is technically challenging	^196^
Chromatin isolation by RNA purification (ChIRP-seq)	Determine the genomic loci that interact with a specific RNA transcript	High-throughput tool to study the RNA-chromatin interactions, but not suitable for the relatively low-abundant eRNAs	^198,199^
Comprehensive identification of RNA-binding proteins by mass spectrometry (ChIRP-MS)	Identify proteins that interact with a specific RNA transcript	Detect RNA-binding proteins (RBPs) with high accuracy in their natural cellular environment, but require careful design of biotin-labeled probes and a substantial amount of starting material	^203–205^
RNA pull-down	Identify proteins that interact with a specific RNA transcript	Widely used biochemical technique, but may not fully recapitulate the native cellular environment	^313,314^
RNA Antisense Purification (RAP)	Determine the genomic loci and protein partners that interact with a specific RNA transcript	Selective purification of endogenous RNA complexes from cell extracts for DNA and protein identification, but requires longer (~90 nt) antisense probes	^208^
Capture hybridization analysis of RNA targets (CHART)	Determine the genomic loci that interact with a specific RNA transcript	Enrich endogenous RNAs with their DNA and protein targets from reversibly cross-linked chromatin extracts, but may have a lower yield compared to RAP or ChIRP	^205^
individual-nucleotide resolution UV-crosslinking and immunoprecipitation (iCLIP)	Study RNA-protein interactions at the single-nucleotide level	Identify the interactions between eRNAs and proteins in their native context within cells, but require a sufficient amount of starting material for effective immunoprecipitation and RNA recovery	^209^
CRISPR-assisted RNA–protein interaction detection (CARPID)	Identify proteins that interact with a specific eRNA	Capture interactions in their native environment, but indirectly interacting proteins may also be labeled	^142,143,210^
Selective 2’-hydroxyl acylation analyzed by primer extension and sequencing (SHAPE-Seq)	RNA structure characterization	Enable high-throughput analysis of RNA secondary structure, but require high RNA quality and abundance	^318,319^

##### Identification of interaction partners

In addition to directly manipulating RNAs, understanding their interactions with protein and DNA partners is equally crucial for elucidating their functions. Chromatin–associated RNA sequencing (ChAR–seq) is a widely used approach to map all RNA–DNA contacts across genome maps.^196^ Since most eRNAs are chromatin-bound transcripts,^86,197^ enriching for chromatin-associated RNAs can improve the detection and characterization of low-abundance eRNAs. Chromatin isolation by RNA purification sequencing (ChIRP–seq) also permits the identification of both *trans* and *cis* genomic loci that interact with a target RNA of interest.^198,199^ This method employs multiple tiling antisense oligos to capture the target RNA–chromatin complexes.^200,201^ Although this method has been applied to map the DNA-binding regions of nuclear lncRNAs,^199,201,202^ it has not been widely used to characterize eRNA-bound DNA regions,^202^ likely owing to the low abundance of eRNAs, which limits their efficient capture by ChIRP probes. The *Chang* group also established an approach called comprehensive identification of RNA-binding proteins by mass spectrometry (ChIRP–MS), which permits the identification of candidate proteins interacting with any target RNA of interest.^203,204^ This method further complements ChIRP-seq by providing functional insights into the protein interactors of nuclear lncRNAs. Similarly, capture hybridization analysis of RNA targets (CHART)^205^ and RNA antisense purification (RAP)^206,207^ can be used in conjunction with DNA sequencing and mass spectrometry to identify DNA-binding regions and protein partners of specific RNA transcripts. Alternatively, RNA-interacting proteins can be captured through conventional RNA pull-down and, more precisely, individual–nucleotide-resolution crosslinking and immunoprecipitation (iCLIP), which can pinpoint the RBP-binding sites on RNA at the nucleotide level.^208,209^ CRISPR–assisted RNA–protein interaction (CARPID) leverages the CRISPR–Cas13d RNA-targeting system for covalent labeling of proteins in close proximity to the RNA of interest in their native cellular context, thereby enabling in situ identification of RNA–protein interactions within specific subcellular compartments.^142,143,210^ As discussed above, the low abundance and inherent instability of eRNAs pose significant challenges for identifying their interacting DNA regions and associated proteins. Therefore, further advancements in techniques capable of capturing these low-abundance transcripts are essential for improving the identification of their genomic and protein interaction partners.

These advanced methodologies provide a robust and versatile toolkit for the detection and functional investigation of eRNAs (Table 2). Notably, with increasing interest in eRNAs, massive amounts of sequencing resources and specialized databases have become available. Table 3 includes the major databases that have been developed to provide comprehensive annotations and analyses of eRNAs in terms of their expression landscape, regulatory network, and potential functions.^24,25,48,211–215^

**Table 3:** Overview of databases for eRNA prediction

Database	Description	Refs.
ENCODE	The ENCODE project provides comprehensive annotations of the human genome, including the identification of gene-regulatory elements, such as enhancers, promoters, insulators, and non-coding RNAs, including eRNAs (https://www.encodeproject.org/)	^49^
eRic (eRNA in Cancer)	A database for studying the roles of eRNAs in cancer by collecting the whole eRNA expression profile across TCGA samples as well as the eRNA clinical features, eRNA target genes and eRNA drug response (https://hanlaboratory.com/eRic/)	^24^
CancereRNAQTL	A database that systematically explores the effects of single-nucleotide polymorphisms (SNPs) on eRNA expression (http://canernaqtl.whu.edu.cn/#/)	^25^
HeRA	A user-friendly data portal for searching, browsing, and downloading the eRNA expression profile, trait-related eRNAs, and eRNA co-expression networks (https://hanlab.uth.edu/HeRA/)	^211^
Animal-eRNAdb	A user-friendly database for the browsing, searching, and downloading of eRNA-related information in multiple species (https://gong_lab.hzau.edu.cn/Animal-eRNAdb/)	^212^
eRNAbase	A database that stores massive available resources of human and mouse eRNAs and provides comprehensive annotations and analyses for eRNAs (https://bio.liclab.net/eRNAbase/index.php)	^213^
eRNA-IDO	A computational platform for the identification, interactome discovery, and functional annotation of human eRNAs (https://bioinfo.szbl.ac.cn/eRNA_IDO/)	^214^

## Functions and mechanisms of eRNAs in gene regulation

Although eRNA production was initially regarded as a random transcriptional event due to the open chromatin status at enhancer regions, later studies have revealed the potential and functional importance of eRNAs in controlling transcriptional programs.^22,197,216^ Increasing evidence has shown that most short, unstable eRNAs function *in cis* to activate the transcription of adjacent genes in the genome.^45,59,79,80,104,217^ However, multiple studies have also demonstrated that long, polyadenylated eRNAs can function in trans to affect genes on a different chromosome from where they are produced.^202,218,219^ Future investigations to better understand how eRNA features impact their modes of action may help to predict whether a given eRNA functions *in cis* or in trans.

### Trapping transcription factors

**Fig. 4:**
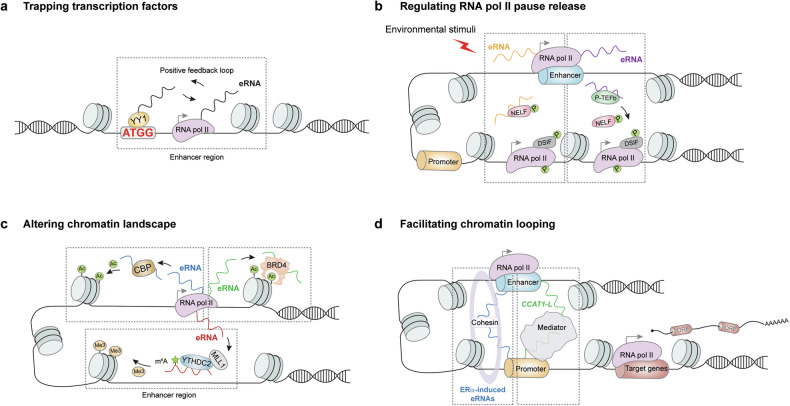
Mechanisms of eRNAs in gene regulation. Schematic diagram depicting the common roles of eRNAs in trapping transcription factors to enhancer regions (**a**), regulating RNA pol II pause release at gene promoter-proximal regions (**b**), influencing the chromatin landscape through altering histone modifications (**c**), and facilitating chromatin looping between enhancers and promoters (**d**). RNA pol II RNA polymerase II, *CCAT1-L*, *colorectal cancer-associated transcript 1, the long isoform*, ERα estrogen receptor α, ORF open reading frame, YY1 Yin-Yang 1, P-TEFb positive transcription elongation factor, NELF negative elongation factor, CBP CREB-binding protein, BRD4 bromodomain containing 4, YTHDC2 YTH *N*^*6*^-methyladenosine RNA binding protein C2, m^6^A *N*^6^-methyladenosine, MLL1 mixed-lineage leukemia protein-1, p phosphorylation, ac acetylation, me3 trimethylation, DSIF DRB sensitivity-inducing factor

eRNAs can trap transcription factors in enhancer regions to maintain enhancer activity. The transcription factor Yin–Yang 1 (YY1) binds to both active enhancer DNA elements and enhancer-derived nascent eRNAs throughout the genome of embryonic stem cells (ESCs).^110^ Inhibition of transcription elongation or RNase A-directed global RNA degradation greatly mitigates YY1 occupancy at enhancer regions. In contrast, dCas9-based tethering of a 60–nucleotide core RNA fragment derived from the *Arid1a* promoter to various enhancer loci promotes YY1 binding at these elements.^110^ This transcription factor trapping model suggests that YY1 binds to and activates enhancer transcription to produce eRNAs that, in turn, interact with and reinforce YY1 local occupancy, which establishes a positive feedback loop to increase YY1-induced enhancer activity^110^ (Fig. 4a). This model highlights that the eRNA-mediated transcription factor trapping mechanism may stabilize the gene transcription program.

### Regulating the pause release of RNA pol II

Another important mechanism by which eRNAs impact the transcription program is regulating RNA pol II pause release at gene promoter-proximal regions. RNA pol II pausing upon transcription initiation is a genome-wide mechanism to regulate the transcription elongation rate.^83^ Negative elongation factor (NELF) directly binds to RNA Pol II to induce pausing.^220^ P-TEFb phosphorylates the negative elongation factor DRB sensitivity-inducing factor (DSIF), converting it into a positive elongation factor, and phosphorylates NELF, leading to its dissociation from RNA polymerase II.^220–222^ Neuronal activity triggers the production of eRNAs, and their knockdown via shRNA or ASOs leads to reduced expression of their *in cis* target mRNAs.^75,223^ Mechanistically, eRNAs bind to the NELF complex, as demonstrated by both in vivo and in vitro binding assays, and function as molecular decoys to promote its dissociation from paused RNA pol II^75,223^ (Fig. 4b). The released RNA pol II enters an active elongation stage in target gene bodies to facilitate mRNA expression in neurons.^75,223^ Androgen receptor (AR)-induced *prostate-specific antigen* (*PSA*) *eRNA*, which is also known as *kallikrein-related peptidase 3 (KLK3) eRNA* (*KLK3e)*, binds and activates P-TEFb to phosphorylate the DSIF/NELF complex.^220,221^ This phosphorylation event facilitates the release of paused RNA pol II, promoting productive transcription elongation in castration-resistant prostate cancer (CRPC) cells^220,221^ (Fig. 4b). siRNA-mediated or ASO-mediated *PSA eRNA* knockdown reduces the level of Ser2-phosphorylated RNA pol II at the *PSA* promoter and inhibits the growth of CRPC cells.^220,221^ Given that the release of RNA pol II from pausing is required for the production of eRNAs, which in turn facilitate RNA pol II pause release, eRNAs are likely contributors, rather than primary determinants, to this process. Environmental stimuli, such as neural activity and AR signaling, as discussed above, may serve as critical triggers that initiate the eRNA-driven positive feedback loop (Fig. 4b).

### Altering the chromatin landscape

eRNAs can interact with histone modifiers and histone modification readers to augment local enhancer activity. The histone acetyltransferase CBP can bind to enhancers to facilitate histone acetylation.^224,225^ Photoactivatable ribonucleoside-enhanced crosslinking and immunoprecipitation (PAR-CLIP) identified a broad set of eRNAs that directly interact with CBP and stimulate its acetyltransferase activity, as shown by both in vitro assays and in vivo studies in mouse embryonic fibroblasts (MEFs).^226^ As a result, CBP-mediated histone acetylation, such as H3K27ac, is promoted by eRNAs at enhancer regions, which contributes to enhancer activities for target gene transcription^226,227^ (Fig. 4c). A recent study revealed that eRNAs can also indirectly bind to histone modifiers.^135^ m^6^A-modified eRNAs produced from super-enhancers are recognized by the m^6^A reader YTH *N*^*6*^-methyladenosine RNA binding protein C2 (YTHDC2), which interacts with H3K4 methyltransferase mixed-lineage leukemia protein-1 (MLL1) to increase H3K4me3 modification at local enhancers in PDAC cells^135^ (Fig. 4c). As a result, the chromatin accessibility of these super-enhancers is facilitated by m^6^A-eRNAs to activate downstream oncogene expression.^196^ BRD4 recognizes and binds to histones with acetylation at enhancer regions to maintain enhancer activity.^228^ Ultraviolet-cross-linked RNA immunoprecipitation (UV-RIP) assays revealed that BRD4 binds to a large set of tumor necrosis factor α (TNF-α)-induced eRNAs.^117^ Electrophoretic mobility shift (EMSA) assays and in vitro pull-down experiments confirmed direct interactions between the BRD4 protein and two representative eRNAs. Furthermore, shRNA-mediated knockdown of these two eRNAs alleviated BRD4 enrichment at their corresponding enhancer regions. Analysis of BRD4 truncation mutants demonstrated that BRD4-eRNA interactions are mediated by BRD4 tandem bromodomains. These findings support a model in which eRNAs associate with BRD4 to reinforce its retention at local enhancers and thereby form an epigenetic positive feedback loop to activate the expression of nearby TNF-α-induced inflammatory genes in colorectal cancer cells^117^ (Fig. 4c). Our recent study revealed a transforming growth factor (TGF)-β-induced *SNAIL family transcriptional repressor 1 (SNAI1)* eRNA (*SNAI1e)* that interacts with BRD4 to promote its binding to H3K27ac-enriched enhancer regions. Loss-of-function analyses using ASOs, Cas13d, siRNAs, CRISPRi and Cas9-mediated enhancer depletion demonstrated that *SNAI1e* knockdown inhibits the transcription of the nearby *SNAI1* gene in breast cancer cells. In contrast, CRISPR display-directed *in cis* overexpression of *SNAI1e* increases BRD4 abundance at the local enhancer and promotes *SNAI1* expression.^229^ These studies highlight the contribution of eRNAs to the local enhancer landscape through interactions with histone modifiers and chromatin modification readers.

### Facilitating enhancer‒promoter looping

Physical 3D chromatin loops between enhancers and promoters are stabilized by architectural protein complexes such as CCCTC-binding factor (CTCF), Cohesion, and Mediator.^230–234^ As another layer of enhancer regulation, eRNAs have been implicated in enhancer‒promoter looping. The first evidence that several ERα-induced eRNAs directly interact with the cohesion core component RAD21 and structural maintenance of chromosomes 3 (SMC3) to strengthen ERα-induced enhancer–promoter looping in breast cancer cells was reported a decade ago^86^ (Fig. 4d). In another study, the 5200 nt eRNA *CCAT1-L* (*Colorectal Cancer Associated Transcript 1, the Long isoform*) was characterized from an enhancer locus upstream of the proto-oncogene *MYC*.^235^ ASO-mediated *CCAT1–L* depletion suppresses *MYC* transcription in colorectal cancer cells.^235^ These results were further consolidated via the use of transcription activator-like effector nucleases (TALENs) to mediate the insertion of either a cytomegalovirus (CMV) promoter for *in cis* overexpression or a double poly(A) site cassette for *in cis* knockdown. Mechanistically, *CCAT1–L* binds CTCF to maintain the CTCF-directed chromatin looping between the enhancer and promoter of *MYC*.^235^ A recent study demonstrated that *−5* *kb Nanog super-enhancer antisense eRNA* (*−5KNAR*) interacts with RAD21 to stabilize the cohesion complex at the *Nanog* locus, thereby maintaining enhancer–promoter looping.^236^ siRNA-mediated *−5KNAR* depletion disrupts this looping and promotes DNA methylation at the *Nanog* promoter, consequently facilitating the differentiation of mouse ESCs.^236^ Moreover, eRNAs can modulate chromatin looping by interacting with Mediator components such as Mediator complex subunit 1 (MED1) in prostate cancer cells.^218^

### DNA:eRNA R-loop structures in gene regulation

**Fig. 5:**
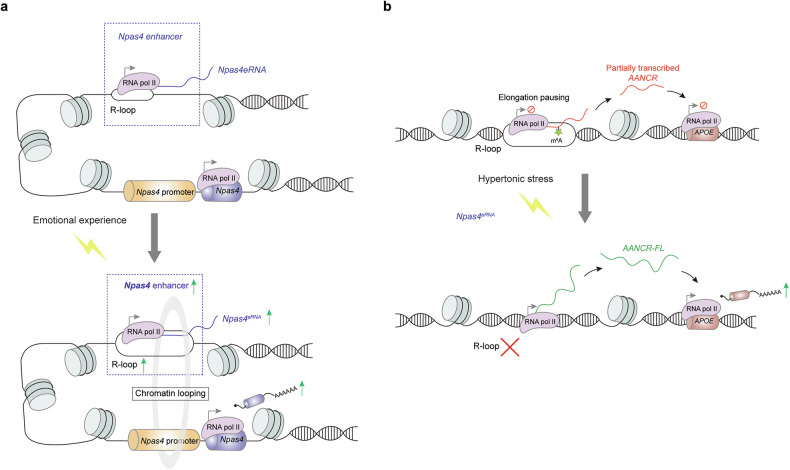
DNA:eRNA R-loop structures in gene regulation. Three-stranded DNA:eRNA hybrid R-loop structures activate or suppress gene transcription, respectively. The former is mediated through facilitating chromatin looping between enhancers and promoters (**a**), whereas the latter is achieved by inducing RNA pol II pausing during eRNA transcription elongation (**b**). RNA pol II RNA polymerase II, *Npas4 neuronal PAS domain protein 4*, *APOE apolipoprotein E*, *AANCR APOE-activating non-coding RNA*, FL full length

R-loops are three-stranded DNA:RNA hybrid structures frequently formed during gene transcription.^237^ Accumulative evidence has revealed that R-loop structures either activate or suppress gene transcription.^238,239^ A recent study demonstrated that emotional stimuli trigger the transcription of a highly conserved eRNA*, neuronal PAS domain protein 4* (*Npas4*)^*eRNA*^, which produces an R-loop structure at the local *Npas4* enhancer region.^240^ The R-loop facilitates chromatin looping between the *Npas4* enhancer and its proximal promoter to induce rapid *Npas4* expression in mouse brain tissues^240^ (Fig. 5a). Genomic locus-specific disruption of R-loops via a dCas9-RNase H1 fusion protein, but not its enzyme-dead mutant, attenuates the promoting effect of *Npas4*^*eRNA*^ on *Npas4* expression.^240^ In contrast, R-loop formation during eRNA transcription can also suppress gene expression. R-loop structures are formed at m^6^A sites on *apolipoprotein E* (*APOE*)*-activating non-coding RNA* (*AANCR*). Analysis of RNA-seq and chromatin immunoprecipitation sequencing (ChIP-seq) data revealed that m^6^A modification of eRNAs may be associated with RNA pol II pausing and transcriptional inactivation of their downstream genes.^241^ In response to hypertonic stress, the R-loops are resolved to enable full-length *AANCR* transcription and the subsequent rapid expression of *APOE* in renal proximal tubule cells^241^ (Fig. 5b).

### Involvement of eRNAs in transcriptional condensate formation

**Fig. 6:**
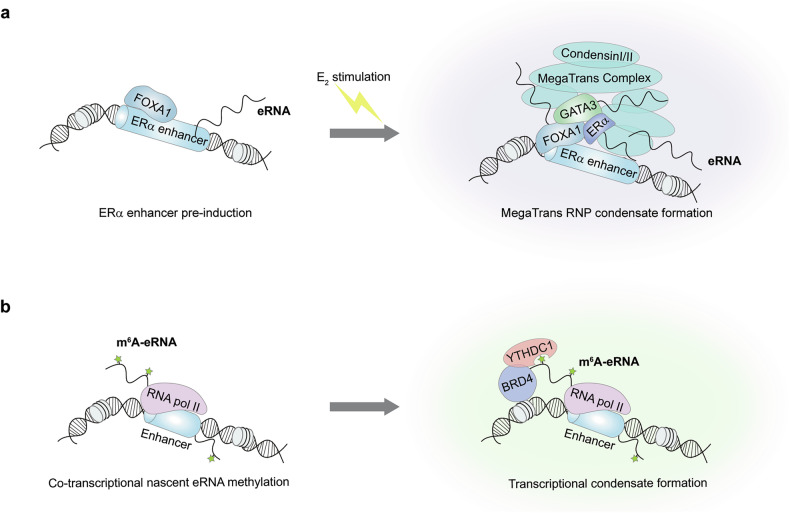
Involvement of eRNAs in transcriptional condensate formation. **a** eRNAs derived from ERα-responsive enhancers activate gene transcription by facilitating the recruitment of transcription factors to their local enhancer regions and the formation of phase-separated transcriptional condensates. **b** m^6^A-marked eRNAs contribute to the formation of BRD4-enriched coactivator condensates. RNA pol II RNA polymerase II, GATA3 GATA binding protein 3, FOXA1 forkhead box A1, ERα estrogen receptor α, E_2_ estradiol, YTHDC1 YTH *N*^*6*^-methyladenosine RNA binding protein C1, BRD4 bromodomain containing 4; m^6^A, *N*^6^-methyladenosine

Intracellular membrane-less organelle assembly is mediated by liquid–liquid phase separation (LLPS), which spontaneously demixes a homogeneous solution into phases with high and low concentrations.^242,243^ LLPS facilitates transcriptional condensate formation, a key process to assemble and compartmentalize the transcription machinery at enhancer regions for enhancer activation.^244,245^ Transcriptional condensate formation is mediated by transcription factors harboring intrinsically disordered regions (IDRs).^246^ Recent studies have shown that eRNAs can promote transcriptional condensate formation to facilitate enhancer activation.^136,247^ Under unstimulated conditions, FOXA1 binds to ERα-responsive enhancers to maintain minimal eRNA transcription. In response to acute estrogen signaling, a transcriptional condensate containing eRNAs and transcription factors with IDRs is formed as a ribonucleoprotein (RNP) complex at ERα-responsive enhancers in breast cancer cells^247^ (Fig. 6a). ASO-mediated depletion of *TFF1e* eRNA derived from the ERα-responsive enhancers diminishes the recruitment of transcription factors to their local enhancer regions and the formation of phase-separated transcriptional condensates.^247^ Mixing in vitro-transcribed *TFF1e* with purified ERα protein shortens the recovery time in fluorescence recovery after photobleaching (FRAP) experiments, suggesting that *TFF1e* facilitates the formation of ERα condensates.^247^ Another study revealed the contribution of m^6^A-modified eRNAs to the formation of BRD4-enriched coactivator condensates.^136^ Many nascent long and stable eRNAs are co-transcriptionally modified with m^6^A in breast cancer cells.^136^ m^6^A-marked eRNAs are recognized and bound by the m^6^A reader YTH *N*^*6*^-methyladenosine RNA binding protein C1 (YTHDC1), which recruits BRD4 to local enhancers, promoting phase separation and transcriptional condensate formation.^136^ Removal of m^6^A marks on selected eRNAs via the dCas13d–FTO system (as discussed in the sections above and below) suppresses YTHDC1 recruitment to enhancer regions and inhibits the formation of transcriptional condensates.^136^ This study unravels novel crosstalk between chemically modified eRNAs, their reader proteins, and transcriptional coactivators through condensates to facilitate local enhancer activity^136^ (Fig. 6b).

### *Trans* regulatory role of eRNAs in gene expression

**Fig. 7:**
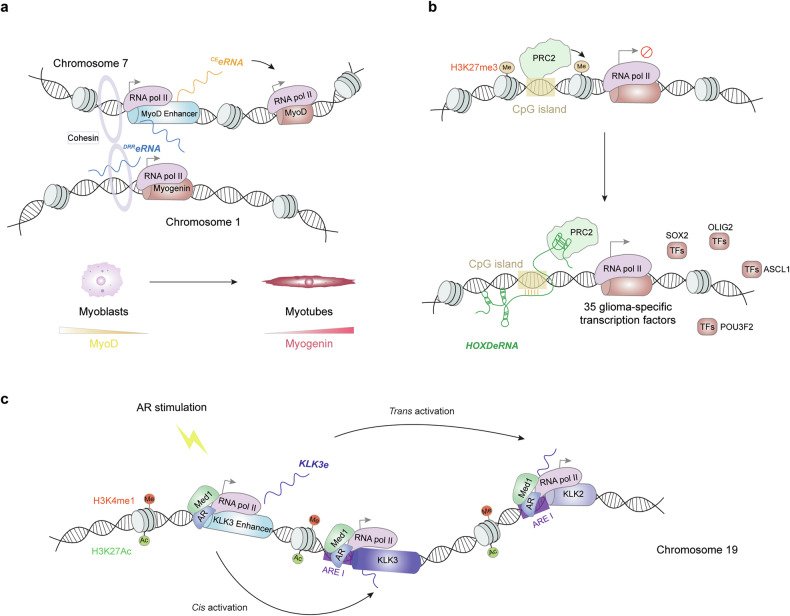
*Trans*-regulatory role of eRNAs in gene expression. **a** Dual roles of eRNAs transcribed from the enhancer region of *MyoD*. ^*CE*^*eRNA* acts *in cis* to increase the expression of the adjacent *MyoD* gene, while ^*DDR*^*eRNA* functions in trans to promote the transcription of *Myogenin* through interacting with and recruiting the cohesion complex to the *Myogenin* locus. These events promote myoblast differentiation through the establishment of a feedforward loop that reinforces the myogenic transcriptional program. **b** *HOXDeRNA* containing a distinct RNA quadruplex structure directly binds to PRC2-covered CpG islands and PRC2, leading to the activation of glioma driver gene expression in a genome-wide manner. **c** AR-induced *KLK3e* acts *in cis* to activate the expression of its neighboring gene, *KLK3*. Additionally, *KLK3e* functions in trans to increase long-distance transcriptional activation of *KLK2* through AR-dependent enhancer–promoter looping. RNA pol II RNA polymerase II, MyoD myogenic differentiation 1, ^*DDR*^*eRNA distal regulatory region eRNA*, ^*CE*^*eRNA* *core enhancer RNA*, H3K27me3 H3 lysine 27 trimethylation, TF transcription factor, ORC2 origin recognition complex subunit 2, SOX2 SRY-box transcription factor 2, OLIG2 oligodendrocyte transcription factor 2, ASCL1 achaete-scute family bHLH transcription factor 1, POU3F2 POU class 3 homeobox 2, H3K4me1 H3 lysine 4 monomethylation, H3K27ac H3 lysine 27 acetylation, AR androgen receptor, *KLK3e kallikrein-related peptidase 3 (KLK3) eRNA*, KLK2 Kallikrein-related peptidase 2, Med1 Mediator 1, ARE androgen response element

Although eRNAs have been well documented to regulate neighboring gene expression *in cis* through various mechanisms, as discussed above, eRNAs can also impact long-range gene expression in a *trans* manner. The enhancer region of *myogenic differentiation 1* (*MyoD*) produces two eRNAs with distinct regulatory roles.^219^ Core enhancer RNA (^*CE*^*eRNA*) acts *in cis* to increase the expression of the adjacent *MyoD* gene. In contrast, ASO-mediated and CRISPRi-mediated loss-of-function analyses revealed that ^*DRR*^*eRNA* functions in trans to promote *Myogenin* transcription through interacting with the *Myogenin* genomic locus — as demonstrated by ChIRP–seq — and recruiting the cohesin complex to this site.^219^ These events promote the differentiation of myotubes by establishing a feedforward loop that reinforces the myogenic transcriptional program^219^ (Fig. 7a). The CpG islands in the promoters of genes encoding multiple key glioma transcription factors are bound by Polycomb repressive complex 2 (PRC2), which deposits trimethylated lysine 27 on histone H3 (H3K27me3) to repress gene transcription.^248,249^ *HOXDeRNA* is selectively recruited to PRC2-covered CpG islands, as demonstrated by the co-localization of *HOXDeRNA* ChIRP-seq signals with PRC2 occupancy, indicated by H3K27me3 and EZH2 ChIP–seq coverage.^202^ Mechanistically, *HOXDeRNA* acts as a decoy to interact with and remove the PRC2 component Enhancer Of Zeste 2 (EZH2) from the promoters of glioma driver genes across the genome, resulting in the transformation of astrocytes into glioma cells^202^ (Fig. 7b). A bidirectionally transcribed eRNA, *KLK3e*, approximately 2,200 nucleotides in length, functions *in cis* to activate the expression of its neighboring gene, *KLK3*.^218^ Interestingly, *KLK3e* also acts in trans to increase long-distance transcriptional activation of AR-regulated genes, including *KLK2*, through the AR-dependent enhancer–promoter looping complex in prostate cancer cells.^218^ siRNA-mediated *KLK3e* knockdown inhibited *KLK2* promoter activity, as demonstrated by ChIP analysis. Moreover, *KLK3e* ectopic expression restored this inhibitory effect in luciferase reporter assays in which *KLK2* promoter activity was measured^218^ (Fig. 7c). This study proposes a model in which a single eRNA can function both *in cis* and in trans to regulate gene expression, highlighting the versatility and complexity of eRNA mechanisms in transcriptional regulation.

## The role of eRNAs in health

The role of eRNAs in various normal physiological biological processes has emerged as crucial, adding an additional layer of complexity to the dynamic gene regulatory networks in normal cells.^219,250–252^

For example, eRNAs are essential for maintaining cardiomyocyte (CM) homeostasis. *NK2 homeobox 5* (*Nkx2-5*) encodes a master transcription factor for properly differentiating cardiomyocytes during heart development.^253,254^ Notably, both the positive and negative DNA strands of the *Nkx2-5* enhancer can transcribe eRNAs with opposing effects. *IRENE-SS* is encoded by the same strand (SS) of the Intergenic Regulatory Element *Nkx2-5* Enhancers (IRENEs), whereas *IRENE-div* is derived from the divergent direction (div). *IRENE-SS* acts as a canonical promoter to increase *Nkx2-5* transcription by recruiting NKX2-5 to its own promoter and enhancer. In contrast, *IRENE-div* increases the local binding of HDAC sirtuin 1 (SIRT1) to the *Nkx2-5* local enhancer, thereby silencing its transcription. Both transcripts must be under proper regulation during the differentiation of human-induced pluripotent stem cells (hiPSCs) into the CM lineage in vitro.^250^

The specification and differentiation of skeletal stem muscle cells into mature myofibers are regulated primarily by a group of myogenic regulatory factors (MRFs), including MyoD, myogenic factor 5 (Myf5), myogenin and myogenic factor 6 (Myf6).^255,256^ As previously mentioned, the enhancer regions of *MyoD* can produce ^*CE*^*eRNA* and ^*DDR*^*eRNA*, both of which play pivotal roles in myogenesis. During muscle differentiation, ^*CE*^*eRNA* promotes adjacent *MyoD* expression through enhancing RNA pol II occupancy and residency at *MyoD*. ^*DRR*^*eRNA* subsequently activates *Myogenin* expression by facilitating local chromatin accessibility, thereby establishing a feedforward loop that reinforces the myogenic transcriptional network.^219,251^ Notably, Tsai P.F. et al. revealed that ^*DRR*^*eRNA* directly interacts with the cohesin complex subunit SMC3 and then recruits the cohesin complex to the *Myogenin* locus. Depletion of either cohesin or ^*DRR*^*eRNA* leads to reduced chromatin accessibility, impaired *Myogenin* activation and defective muscle cell differentiation.^219^ Notably, the cohesin complex is equally expressed in undifferentiated cells but fails to be actively loaded onto the *Myogenin* locus. This observation highlights the *trans*-regulatory role of ^*DRR*^*eRNA* in properly loading and maintaining the cohesin complex during myogenic differentiation.

In obesity research, a comprehensive transcriptome study by RNA-seq in adipocytes demonstrated that the eRNA *Lnc-leptin*, transcribed from an enhancer region upstream of the *leptin* (*Lep*) gene, regulates *Lep* expression by acting as a bridge to increase the interaction between the *Lep* promoter and enhancer.^252^ The results from multiple independent loss-of-function approaches indicate the necessary, but not sufficient, role of *Lnc-leptin* in promoting *Lep* expression and adipogenesis. Although the depletion of *Lnc-leptin* during adipogenesis results in significant reductions in lipid accumulation and the expression of mature adipocyte markers, the formation of mature adipocytes in *ob*/*ob* mice is not impaired, suggesting that *Lnc-leptin* may regulate adipogenesis through a *Lep*-independent mechanism.

SWI/SNF chromatin remodelers are required for the activity of certain enhancers that are important for cell identity.^257–259^ Recently, Saha D. et al. revealed that the AT-hook domain of Brg1 preferentially binds to *cis*-acting eRNAs, leading to the global recruitment of SWI/SNF to cell type-specific enhancers.^260^ Consequently, SWI/SNF regulates the transcription of cell lineage priming-related genes through the recruitment of MLL3/4, p300/CBP, and the Mediator complex. These findings suggest the significant role of eRNAs in early mammalian development, particularly in the transition from a naive pluripotent state toward cell lineage priming.^260^

Taken together, eRNAs have emerged as key regulators of various physiological processes, such as cardiomyocyte homeostasis, skeletal muscle differentiation, adipogenesis, and early embryonic development. A deeper understanding of their diverse functions and mechanisms will offer valuable insights into the complexity of gene regulation networks in normal cells.

## The role of eRNAs in diseases

Growing evidence has shown the importance of enhancer malfunction in tumorigenesis, where both genetic mutations and epigenetic alterations of enhancer elements drive the initiation and progression of cancers.^261–263^ Accordingly, aberrant expression of enhancer-derived eRNAs is strongly associated with the dysregulation of cancer-related genes and the activation of abnormal cellular responses.^24,25,218,235,262,264–266^ Table 4 lists the key eRNAs implicated in cancer.

**Table 4:** List of eRNAs implicated in cancer

Disease Type	eRNA	Biological function and mechanisms of action	Expression (tumor vs normal)	Prognosis	Refs
Basal cell carcinoma	*ACTRT1e*	Mutations in *ACTRT1e* impair the expression of *ACTRT1*, thereby activating Hedgehog signaling.	Not determined	Not determined	^381^
Bladder cancer	*HPSE eRNA*	*HPSE eRNA* promotes cancer cell proliferation, migration, and invasion in vitro and in vivo. It binds to hnRNPU to promote its interaction with p300, and thereby facilitates promoter-enhancer looping of *HPSE*.	Up	Worse	^159^
Bladder cancer	*P2RY2e*	Estrogen-induced *P2RY2e* promotes cancer cell survival, migration, and invasion.	Up	Not determined	^260^
Breast cancer	*DSCAM-AS1*	*DSCAM-AS1* is upregulated in breast cancer, prostate cancer, and lung adenocarcinoma. In breast cancer, *DSCAM-AS1* interacts with YBX1 and influences the recruitment of YBX1 at the promoter regions of *FOXA1* and *ESR1* (encoding ERα).	Up	Worse	^382^
Breast cancer	eRNAs derived from p53-bound enhancer regions	p53-induced eRNAs produced from p53-bound enhancer regions are required for transcriptional activation of their corresponding neighboring genes and an efficient p53-dependent cell-cycle arrest.	Not determined	Not determined	^270^
Breast cancer	*NET1e*	*NET1e* promotes cancer cell growth and drug resistance by enhancing *NET1* expression.	Up	Worse	^24^
Colorectal cancer	*CCAT1* (*CCAT1-L*)	*CCAT1* interacts with CTCF to promote long-range chromatin looping between the promoter and enhancer of *MYC*. *CCAT1* expression predicts BET inhibitor sensitivity in colorectal cancer.	Up	Worse	^235,265,383^
Esophageal and head and neck squamous cell carcinomas	*LINC01503*	*LINC01503* is upregulated in squamous cell carcinomas. It promotes cell proliferation, migration, invasion, and growth of xenograft tumors.	Up	Worse	^384^
Gastric cancer	*BIR3C eRNA*	*H. pylori*-induced *BIR3C eRNA* promotes *H. pylori*-induced *BIR3C* and *cIAP2* expression to inhibit cancer cell apoptosis.	Not determined	Not determined	^385^
Hepatocellular Carcinoma	*HCCL5*	*HCCL5* is upregulated in HCC. It promotes the growth, invasion, and metastasis of HCC cells both in vitro and in vivo.	Up	Worse	^386^
Hepatocellular Carcinoma	*LINC01089*	*LINC01089* is upregulated in HCC. It regulates hnRNPM-controlled *DIAPH3* mRNA splicing in HCC.	Up	Worse	^387^
Lung adenocarcinoma	*LINC00880*	*LINC00880* regulates the CDK1/PRDX1 axis to sustain the malignancy of lung adenocarcinoma.	Up	Worse	^388^
MLL-rearranged leukemia	*SEELA*	*SEELA* depletion suppresses cell proliferation in vitro and leukemia progression in vivo. Chromatin-bound *SEELA* binds to the K31 amino acid of histone H4 to strengthen the interaction between chromatin and histone modifiers to activate *SERINC2* transcription.	Not determined	Not determined	^389^
Nasopharyngeal carcinoma	*seRNA-NPCM*	*seRNA-NPCM* regulates *NDRG1* and *TRIB1* transcription by facilitating long-distance chromatin interactions between super-enhancers and promoters, thereby promoting metastasis of nasopharyngeal carcinoma.	Up	Worse	^390^
Prostate cancer	*KLK3e*	*KLK3e* facilitates the spatial interaction between the *KLK3* enhancer and the *KLK2* promoter, and enhancing *KLK2* transcriptional activation. *KLK3e* knockdown inhibits cancer cell proliferation.	Not determined	Not determined	^218^
Squamous cell carcinomas	*CCAT1*	*CCAT1* promotes SCC cell proliferation both in vitro and in vivo through activating downstream MEK/ERK1/2 and PI3K/AKT signaling.	Not determined	Not determined	^391^
Pancreatic ductal adenocarcinoma	*MYC-490-kb eRNA*	*MYC-490-kb eRNA* interacts with YEATS2 to augment the association of YEATS2-containing ATAC complexes with *MYC* promoter/enhancer regions and thus increases *MYC* gene expression.	Up	Not determined	^392^
Prostate cancer	*LTFe*	*LTFe* promotes *LTF* transcription by interacting with HNRNPF to facilitate enhancer-promoter interactions. *LTFe* knockdown inhibits ferroptosis and promotes cancer cell growth.	Down	Better	^393^

### eRNAs as biomarkers

Although the overall expression levels of eRNAs are generally lower than those of mRNAs, a subset of eRNAs has demonstrated significant potential as biomarkers for diagnosis and prognosis in clinical settings. Zhang Z. et al. identified a total of 9108 detectable eRNAs (reads per million ≥1) across various human cancers by mapping The Cancer Genome Atlas (TCGA) RNA-seq reads to eRNA regions and revealed cancer type-specific patterns of eRNA expression, indicating the potential utility of eRNA expression signatures for cancer diagnosis.^24^ Additionally, certain differentially expressed eRNAs are correlated with patient survival and other cancer-related clinical features. Examples of such correlations include eRNA expression and survival (e.g., *neuroepithelial cell-transforming gene 1 protein-associated eRNA (NET1e) and serine/threonine kinase TAOK1-associated eRNA (TAOK1e*), cancer subtype (*Engrailed 1-associated eRNA* (*EN1e*)), stage (*CUGBP Elav-Like Family Member 1-associated eRNA (CELF2e)*, grade *(Aph-1 Homolog A, Gamma-Secretase Subunit-associated eRNA (APH1Ae)*, and smoking history *(Scribble Planar Cell Polarity Protein-Associated eRNA (SCRIBe)*.^24^ Consistent with these findings, *Enhancer 22* is significantly correlated with worse patient survival across multiple cancer types.^26^

Programmed death-ligand 1 (PD-L1) is critical in cancer immunotherapy because it modulates immune evasion mechanisms.^267,268^ It inhibits T-cell activation upon binding to its receptor, programmed death-1 (PD-1), on T cells, thereby suppressing the immune response and allowing cancer cells to evade immune surveillance.^268,269^ Interestingly, a strong correlation between the expression of *enhancer 9* (chr9:5580709–5581016) and *PD-L1* has been observed in multiple cancer types.^26^ Deletion of this enhancer strikingly impairs *PD-L1* expression at both the mRNA and protein levels.^26^ These findings highlight the potential of eRNAs as biomarkers for assisting in the design of immunotherapies.

### eRNAs as oncogene activators

eRNAs can contribute to cancer progression by activating oncogene expression. The human colorectal cancer-specific eRNA *CCAT1-L* is actively transcribed from a super-enhancer region upstream of the proto-oncogene *MYC*.^235^ *CCAT1-L* knockdown significantly reduces the transcription of nascent *MYC* mRNA, whereas *CCAT1-L in cis* overexpression enhances *MYC* transcription and thereby promotes colorectal cancer progression.^235^ Mechanistically, *CCAT1–L* interacts with CTCF to promote its binding to the *MYC* locus, facilitating chromatin looping between the *MYC* promoter and its enhancer (as discussed in the above section).^235^ Another study demonstrated that targeting *CCAT1* with the small-molecule BET inhibitor JQ1 markedly reduces *MYC* expression and colorectal cancer cell growth.^265^ Of note, JQ1 treatment shows a more potent inhibitory effect on *MYC* transcription in *CCAT1*^*high*^ cells than in *CCAT1*^*low*^ colorectal cancer cells.^265^ However, the causal role of *CCAT1* in *MYC* activation remains debated, as it is difficult to rule out the possibility that JQ1 directly targets the MYC promoter to inhibit transcription. AR-induced eRNAs (e.g., *KLK3e*) selectively increase AR-induced gene expression in prostate cancer cells.^218^ In addition, CRISPRi screening of eRNA-producing super-enhancers in triple-negative breast cancer identified super-enhancer *SE66* and its cognate eRNA transcript, both of which drive the expression of the nearby gene *podocalyxin-like* (*PODXL*). Specific degradation of *SE66* eRNA results in considerable suppression of target gene expression, as well as a marked inhibition of cell proliferation and migration.^266^

### eRNAs as tumor suppressors

Conversely, eRNAs are also involved in mediating the functions of key tumor suppressor genes. For instance, Melo C.A. et al. detected the induction of thousands of eRNAs following p53 transcription factor activation in breast cancer cells.^270^ Importantly, they identified p53-bound enhancer regions (p53BERs) that produce eRNAs to facilitate the transcription of their target genes and thereby induce p53-dependent cell cycle arrest.^270^ Of note, a follow-up study by the same group demonstrated that, rather than binding and being stimulated by activated p53, the transcription of certain p53-regulated enhancer regions (p53RERs) can be preferentially initiated by a regulatory RNA named the lncRNA activator of enhancer domains (*LED*).^271^ Consequently, the expression of downstream target genes, such as *Cyclin Dependent Kinase Inhibitor 1A* (*CDKN1A*), is activated in breast cancer cells.^271^ These findings add an additional layer to the eRNA regulatory network in cancer development.

More recently, a subgroup of highly interactive enhancers, termed iHUBs, characterized by high BRD4 occupancy and eRNA production, has been identified as key mediators of aberrant transcriptional activation in chemoresistant PDAC.^272^ Deleting iHUB or disrupting iHUB transcription reduces enhancer–promoter interaction (EPI) frequency and attenuates resistance to chemotherapy.^272^ Given that eRNA transcription stabilizes the EPI, these findings further highlight its importance in predicting both acquired and intrinsic chemoresistance in patients. Similarly, eRNA productivity at *Cis*-Regulatory Elements (CREs) is also essential in defining the phenotypic heterogeneity of B-cell precursor acute lymphoblastic leukemia (BCP-ALL) following treatment.^273^

These studies underscore the emerging importance of enhancer transcripts in cancer (Table 4) and suggest that eRNAs could serve as promising therapeutic targets for cancer treatment.

### Roles of eRNAs in cardiovascular diseases

Cardiovascular diseases (CVDs) constitute a group of disorders occurring in the blood circulatory system, including the heart and its associated blood vessels.^274,275^ The improper involvement of eRNAs in cardiac gene regulatory networks often contributes to CVD development.^276–280^ For example, Ounzain S. et al. identified a human super enhancer-associated ncRNA termed *cardiac mesoderm enhancer-associated non-coding RNA* (*CARMEN*) as a crucial regulator of cardiac precursor cell differentiation and cardiovascular pathology in human hearts.^276^ Depletion of *CARMEN* is accompanied by significant downregulation of key cardiac TFs and structural proteins (*Gata4*, *Nkx2-5* and *Myh6*).^276^ Notably, human *CARMEN* isoforms, in particular *CARMEN3*, are upregulated in both idiopathic dilated cardiomyopathy (DCM) and aortic stenosis.^276^ The cardiac fibroblast (CF)-enriched eRNA *Wisp2 super-enhancer–associated RNA (Wisper)* is positively correlated with cardiac fibrosis in both a murine model of myocardial infarction (MI) and heart tissue from human patients with aortic stenosis. In vivo silencing of *Wisper* reduces cardiac fibrosis and improves cardiac function, highlighting its potential as a therapeutic target for mitigating cardiac fibrosis.^277^ Super-enhancer-associated eRNAs, *myosin heavy-chain-associated RNA transcripts* (*myheart* or *Mhrt*), are cardiac-specific and abundant in the adult mouse heart.^278^ Pathological stress activates the Brg1–Hdac–Parp chromatin repressor complex to suppress *Mhrt* transcription.^278^ Restoring cardioprotective *Mhrt* antagonizes Brg1-triggered aberrant gene expression and cardiac myopathy.^278^ Hypoxia-inducible factors (HIFs) are crucial for maintaining oxygen homeostasis and regulating the pathogenesis of various human diseases, including cancer and cardiovascular diseases.^279–283^ Mirtschink P. et al. demonstrated that *HIF1α-activated eRNA* (*HERNA1*) is robustly upregulated in pressure overload–induced heart disease. In vivo inactivation of *HERNA1* attenuates stress-induced cardiac pathogenesis and dramatically improves overall survival in diseased mice.^280^

### Roles of eRNAs in neurological diseases

Neurological diseases encompass a wide range of disorders that affect the structure and function of the brain, spinal cord, and peripheral nerves.^284,285^ Aberrant enhancer activity has been increasingly implicated in the pathogenesis of various neurological diseases.^286,287^ Given that most characterized enhancer regions are limited to model organisms and transformed human cell lines, Yao P. et al. analyzed multiple published datasets to identify a core set of genomic regions with strong evidence of eRNA expression and to explore eRNA‒gene coexpression interactions. They reported that active brain-expressed enhancers (BEEs) are enriched for genetic variants associated with autism spectrum disorder (ASD).^286^ Notably, their analysis also revealed that a substantial proportion of BEE-produced eRNAs (44%) are selectively expressed in the human brain rather than in cultured neurons or astrocytes, underscoring the critical influence of the cellular microenvironment on enhancer activity regulation.^286^ Moreover, additional comprehensive analyses identified 118 differentially transcribed eRNAs in schizophrenia (SCZ) patients compared with controls,^287^ and a total of 77 eRNAs were significantly induced in response to stroke.^288^ Together, these findings indicate the functional significance and clinical potential of eRNAs in neurological diseases.

### Roles of eRNAs in inflammation

Inflammation is a complex biological response triggered by the immune system to counteract harmful stimuli, including pathogens, injured cells, toxic compounds, or irradiation.^289,290^ It also plays an essential role in tumorigenesis.^291^ Emerging evidence suggests that inflammatory signals can induce enhancer activation and eRNA production.^292,293^ Rhanamoun H. et al. reported that, in response to proinflammatory TNF-α signaling, cobinding of tumor-promoting mutant p53 and nuclear factor kappa B (NF-κB) at a cohort of enhancers can induce eRNA synthesis.^103,117^ In turn, eRNAs also serve as important regulatory elements in the inflammatory response process and maintain immune homeostasis.^100,294^ For example, RNA-seq in primary human monocytes revealed a total of 76 differentially expressed eRNAs in response to bacterial lipopolysaccharide (LPS) stimulation. One notable example is the eRNA *IL-1β-eRNA*, which is located downstream of the *IL-1β* gene and whose expression is regulated by the classical proinflammatory transcription factor NF-κB. Crucially, the knockdown of LPS-induced *IL-1β-eRNA* selectively attenuated the transcription and protein release of IL-1β and, to a lesser extent, that of *CXCL8*. This evidence indicates the *cis-*regulatory and *trans*-regulatory roles of eRNAs in the human innate immune response.^100^ Similarly, GRO-seq revealed eRNA transcription events at the *Ccl2* enhancer region in RAW264.7 cells upon inflammatory LPS stimulation. The eRNA transcribed from the *Ccl2* enhancer E region enhances *Ccl2* mRNA transcription by modulating CBP-mediated H3K27ac and facilitating sub-TAD formation via enhancer‒promoter looping. Knockdown of the *Ccl2* enhancer E-derived eRNA in an obese mouse model reduces *Ccl2* mRNA expression and macrophage inflammation in white adipose tissue (WAT) and partially reverses obesity-associated insulin resistance. Because the macrophage-derived chemokine CCL2 is a key mediator of metaflammation, this work implicates the therapeutic potential of targeting eRNA in the context of immune–metabolic disorders.^294^

## Approaches for eRNA therapeutics

**Fig. 8:**
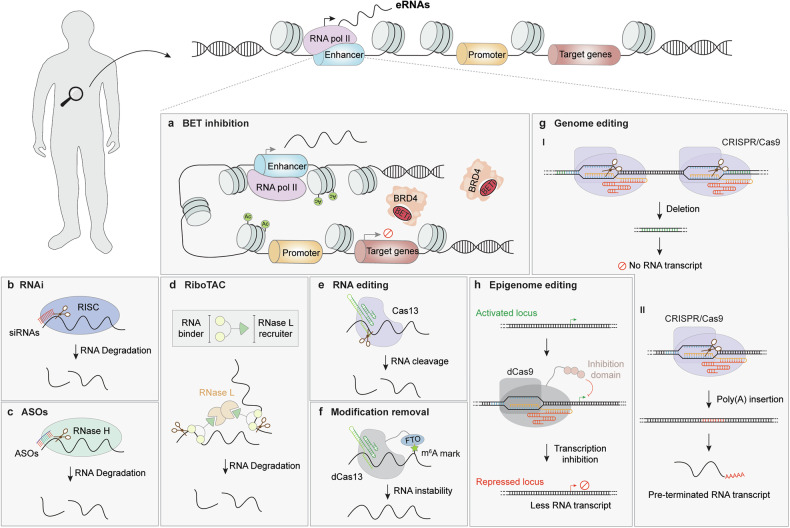
Strategies for targeting eRNAs. **a** BET inhibitors interfere with eRNA transcription by blocking the binding ability of BRD4 to acetylated lysine residues; (**b**-**d**) RNAi, ASOs, and RIBOTACs induce eRNA degradation by recruiting RISC (**b**), RNase H (**c**), and RNase L (**d**), respectively, to the targeted RNA transcripts; **e** RNA editing mediated by CRISPR/Cas13 directly cleaves eRNA transcripts; **f** the CRISPR/dCas13-FTO system reduces RNA stability by removing the m^6^A modification on eRNAs; **g** genome editing tools affect eRNA expression by deleting the entire enhancer region (I) or inserting a poly(A) signal into the targeted region (II); **h** epigenome editing tools inhibit eRNA transcription by altering the chromatin landscape. RNA pol II RNA polymerase II, BET bromodomain and extra-terminal domain, BETi BET inhibitor, BRD4 bromodomain containing 4, RNAi RNA interference, siRNA short interfering RNA, RISC RNA-induced silencing complex, ASO antisense oligonucleotide, RIBOTAC RNA-targeting chimeric small molecule, CRISPR/Cas9 clustered regularly interspaced short palindromic repeats/CRISPR-associated protein 9, dCas9 catalytically inactive Cas9, dCas13 catalytically inactive Cas13, FTO fat mass and obesity-associated protein, m^6^A *N*^6^-methyladenosine, poly(A) polyadenosine signal

eRNAs play crucial roles in regulating gene expression and cellular processes. The dysregulation of eRNAs is often associated with the activation of oncogenes in various cancers.^295,296^ eRNAs derived from overactivated enhancers globally exhibit increased expression in tumor samples compared with their adjacent normal tissues.^24,25^ These observations suggest that eRNAs per se may serve as potential therapeutic targets for cancer therapy. Additionally, the distinctive features of eRNAs, particularly their tissue- and cancer type-specific expression patterns,^24,25^ may permit high cell-type specificity, minimizing adverse on-target side effects, which makes eRNA-targeted therapies appealing and valuable. This section discusses common approaches that have been explored for eRNA targeting (Fig. 8).

### Inhibition of the BET bromodomain

BET family members are epigenetic readers of histone acetylation with broad specificity. BET proteins, consisting of four members (BRD2, BRD3, BRD4, and BRDT), affect chromatin function by interacting with acetylated lysine residues on histone tails.^297^ Notably, BRD4 occupancy at active promoters and enhancers is essential for the recruitment of the elongation factor P-TEFb, which phosphorylates RNA pol II to facilitate lineage-specific gene transcription.^222,298,299^ Indeed, ChIP–seq revealed widespread BRD4 occupancy at the enhancer and promoter regions of active genes.^300^ Hence, BRD4 inhibition by blocking its ability to bind acetylated lysine residues with pan-BETis, such as JQ1,^172^ PFI-1^173^ and I-BET,^174^ is considered a potential therapeutic approach for interfering with eRNA transcription. In agreement with this notion, Kanno T. et al. demonstrated that JQ1 treatment effectively antagonizes BRD4 and impedes the transcription elongation of eRNA transcripts by preventing the release of RNA pol II pause.^109^

Unlike small-molecule inhibitors that occupy the active pocket of BRD4, BRD4-targeting proteolysis-targeting chimeras (PROTACs), such as ARV-825^301^ and dBET1,^302^ leverage the ubiquitin–proteasome system to induce direct intracellular degradation of the BRD4 protein,^303^ offering an alternative approach to targeting BRD4-directed eRNA production. Notably, BRD4 inhibition does not exclusively suppress eRNA expression but also affects the expression of protein-coding genes.^172,173^ Therefore, directly targeting eRNAs for degradation represents a more specific and potentially more effective strategy for eRNA-based therapies.

### Degradation of eRNA transcripts

siRNA and shRNA are widely utilized approaches to manipulate mRNA expression.^304–306^ These strategies exploit the endogenous RNAi machinery, wherein double-stranded RNA (dsRNA) molecules induce posttranscriptional gene silencing by disrupting mRNA stability or translation.^307^ Pioneering work by Lam M.T.Y. et al. demonstrated efficient and selective suppression of two macrophage lineage-associated enhancer RNAs (*matrix mellaloproteinnase 9* (*Mmp9)-eRNA* and *CX3C motif chemokine receptor 1* (*Cx3cr1*)*-eRNA*) via siRNAs, leading to decreased levels of the downstream target genes *Mmp9* and *Cx3cr1*.^58^ Notably, in vivo administration of siRNA potently reduced *Mmp9-eRNA* expression in a sterile peritonitis-induced mouse model.^58^ Moreover, Jiao W. et al. identified *heparanase* (*HPSE*)*-eRNA* as a tumor promoter in gastric cancer cells.^159^ A significant decrease in tumor growth and intratumoral HPSE activity was observed in mouse xenograft models following subcutaneous injection of gastric cancer cells with shRNA-mediated *HPSE-eRNA* knockdown.^159^

In addition to RNAi-directed strategies, chemically modified ASOs have been applied to target eRNAs for degradation.^24,58,86^ ASOs bind to complementary RNA targets, forming RNA‒DNA hybrids that activate nuclear RNA degradation through RNase H-dependent cleavage.^308^ Furthermore, ASOs incorporating a phosphorothioate backbone along with chemically modified residues, such as 2’-O-methoxyethyl (2’-MOE), 2’-O-methyl (2’-OMe), or locked nucleic acids (LNAs), exhibit increased stability and binding affinity to their target sequences.^309,310^ In support of this notion, our recent study revealed that 2’-MOE-modified ASOs potently reduce the expression of *SNAI1e*, resulting in a decrease in the expression of its target gene *SNAI1* and the inhibition of TGF-β-induced epithelial‒mesenchymal transition (EMT), migration, and chemotherapeutic drug resistance in breast cancer cells.^229^ Notably, RNAi/ASO-based strategies face challenges in tissue delivery, including unintended immune activation, poor cellular uptake, and the toxicity associated with delivery vehicles,^311,312^ as discussed in the Concluding remarks and perspectives section.

As an alternative strategy, ribonuclease-targeting chimeras (RIBOTACs) were developed for heterobifunctional small-molecule-directed selective RNA degradation.^313,314^ This approach couples the RNA-binding small molecules to 2’-5’-linked oligoadenylate units (2’-5’ A) to recruit endogenous RNase L. RIBOTACs permit selective recognition and cleavage of the Drosha site within primary microRNA-96 (*pri-miR-96*) or the Dicer site within precursor microRNA-210 (*pre-miR-210*), resulting in significant inhibition of the corresponding transcript expression.^313,314^ To increase drug-likeness, in their follow-up study, they conjugated the RNA-binding compound with a heterocycle to locally activate RNase L, resulting in more potent and long-lasting cleavage activity without triggering global antiviral and/or innate immune responses.^315^ RIBOTACs require the selective binding of small molecules to the RNA of interest; however, merely achieving RNA binding does not necessarily guarantee a desired biological effect. Beyond the sequence per se, the 3D structure of RNA molecules is equally crucial for assessing the druggability of a given RNA target for small molecules.^316^ Single-stranded RNA molecules can fold into highly complex 3D shapes, which determine their functional binding pockets, the accessibility of key structural motifs, and the structural interactions formed at the binding site, such as tertiary interactions or pseudoknots.^317^ Targeting RNA can be achieved either by binding to its functional site or, more effectively, by directly cleaving the RNA, even if binding occurs outside of a functional site. Selective 2’-hydroxyl acylation analyzed by primer extension and sequencing (SHAPE–seq) integrates structure-dependent chemical probing with next-generation sequencing to enable high-throughput characterization of RNA structures.^318,319^ This technique can aid in characterizing the 3D structure of RNA and investigating how drug binding induces structural alterations. Recent advancements in cellular mapping experiments and computational methods have enabled the modeling of 3D RNA structures. Breakthrough tools such as RoseTTAFoldNA,^320^ AlphaFold3,^321^ RhoFold+,^322^ FARFAR2,^323^ MC-Fold/MC-Sym^324^ and iFoldRNA^325^ have provided unprecedented accuracy in predicting RNA conformations. These advancements hold great promise for facilitating the design of RIBOTACs, which enable the selective targeted destruction of RNA molecules, including eRNAs.

Alternatively, eRNAs can be selectively degraded by the type VI CRISPR/Cas system, which specifically cleaves RNA molecules without neutering the genome.^326,327^ This system comprises four distinct subtypes, namely, VI-A, VI-B, VI-C, and VI-D, with each subtype containing a Cas13 effector designated Cas13a (C2c2), Cas13b, Cas13c, and Cas13d, respectively.^17,19,328–330^ In a recent study, we achieved a significant reduction in *SNAI1e* expression through the application of the CRISPR/Cas13d platform in breast cancer cells.^229^

As discussed above, m^6^A profiling studies have captured a substantial proportion of nascent eRNAs marked with m^6^A.^134–136^ These findings demonstrated that m^6^A deposition may influence eRNA stability^136^ and functional interactions.^135,136^ In this context, Lee J.H. et al. developed an m^6^A editor system by fusing a catalytically inactive dCas13d to the m^6^A demethylase FTO.^136^ A significant decrease in *TFF1e* levels was observed when this system was coupled with a specific eRNA-targeting sgRNA.^136^ This approach underscores the potential of erasing m^6^A marks for modulating eRNA expression and function.^331^

### Alternation of genetic information

Direct manipulation of enhancer genomic regions can also regulate the expression of their associated eRNAs. The CRISPR–Cas9 system is one of the most potent and versatile platforms for precise genome editing.^332^ Unlike protein-coding genes, where a single sgRNA is able to terminate protein production through the introduction of frameshift mutations, the genetic information of non-coding elements must be fully, or at least partially, removed to disrupt their activity. For example, paired sgRNAs, together with the Cas9 nuclease, were utilized to delete approximately 650 bp from the 5’ end of the gene, where the lncRNA *Metastasis-Associated Lung Adenocarcinoma Transcript 1* (*MALAT1*) is transcribed, to interfere with *MALAT1* expression in aggressive breast cancer MDA-MB-231 cells.^184,333,334^ However, extensive genomic deletion, especially deletion of key regulatory elements, can influence adjacent gene expression.^333,334^ Alternatively, inserting a transcriptional terminator poly(A) signal sequence into the target non-coding region offers a more precise and controlled strategy to effectively silence gene expression. This approach facilitates premature transcription termination at the target site, thereby minimizing the risk of unintended interference with neighboring genomic elements. Hence, in the same study, the authors generated a distinct *Malat1*-knockout mouse model by inserting a poly(A) sequence 69 bp downstream of the TSS of *Malat1*, equally resulting in the loss of *Malat1* RNA.^184^ Although *MALAT1* is a lncRNA rather than an eRNA, these findings demonstrate a proof-of-principle approach for silencing eRNAs at the genomic level.

### Interference with chromatin accessibility

The active transcription of eRNAs is closely linked to the presence of specific histone modifications at enhancer regions.^62–64^ Editing epigenetic modifications or altering chromatin accessibility enables tunable regulation of gene transcription. Importantly, it avoids triggering endogenous DNA damage and repair pathways, which are the major concerns of conventional genome editing approaches.^335–340^ The CRISPR/Cas9 system can be repurposed by fusing an array of repressive chromatin modifiers, such as the Krüppel-associated box (KRAB) domain, to dCas9.^335^ These effector domains can recruit transcriptional machinery to predefined chromatin loci and then modify histone residues or DNA methylation. However, a longstanding bottleneck in epigenome editing is that epigenetic effectors can only induce transient regulation of gene expression. To address this issue, several co-delivery strategies, such as the co-delivery of three DNA-targeting proteins, each fused separately to KRAB, DNA methyltransferase 3α (DNMT3A), and DNA methyltransferase 3-like (DNMT3L), as well as the co-delivery of DNMT3A–dCas9 and EZH2–dCas9, are reported to permit stable gene silencing.^336,337^ Recently, an even more advanced system, namely CRISPRoff, which fuses a single dCas9 protein to the effector domains of KRAB, DNMT3A and DNMT3L,^341^ has been developed. This system allows highly specific and heritable gene silencing across multiple endogenous loci.^341^ This durable epigenetic memory is obtained through establishing DNA hypermethylation and depositing repressive histone modifications at the targeted loci. However, these regulatory effects may also impact the transcriptional activity of nearby genes or other non-targeted genomic regions. Such interventions require comprehensive therapeutic evaluation to ensure specificity, efficacy, and safety.

Although further research is needed to elucidate the mechanisms of eRNA action, current evidence underscores the promising potential of targeting eRNAs through various therapeutic strategies. By leveraging their roles in cancer progression and tissue-specific expression, eRNA-targeted therapies may pave the way for highly precise and tumor-specific anticancer treatments.

## Concluding remarks and perspectives

To date, despite significant efforts seeking to unravel the functions and mechanisms of eRNAs, the exact role of the eRNA transcription process versus the eRNA transcripts per se in gene regulation remains debatable in certain contexts.^342^ For example, Toll-like receptor 4 (TLR4)-triggered enhancer transcription induces the deposition of H3K4me1 and/or H3K4me2 at a group of enhancers in macrophages.^104,293^ Blocking transcription elongation, rather than targeting eRNA transcripts, potently attenuates the enrichment of H3K4me1 and/or H3K4me2 at these enhancers, leading to weaker enhancer activities.^104^ Another study revealed that depletion of an enhancer-like *cis* element at the non-coding *Lockd* gene locus greatly mitigates the transcription of the neighboring gene *Cdkn1b*.^343^ However, reducing *Lockd* RNA expression by inserting a poly(A) signal downstream of the TSS does not affect *Cdkn1b* transcription.^343^ Additionally, eRNAs most likely function within a domain consisting of interactions between neighboring and distal genomic regions confined to a close 3D space.^344,345^ Nevertheless, it is yet to be determined whether these regulations are achieved in trans or in cis. Addressing these questions will enhance our understanding of the importance of eRNAs in human diseases, including cancer, and aid in evaluating their potential as therapeutic targets.

Second, the human genome is estimated to contain >400,000 enhancers, with ~40,000–65,000 displaying active transcriptional activity.^45,50,84^ However, the biological functionality of eRNAs remains largely unknown owing to their low cellular abundance and inherent instability.^24,346,347^ Recent studies have revealed the widespread presence of eRNAs across a large cohort of tumor samples, identifying a notable subset of clinically relevant eRNAs with cancer type-specific expression patterns.^24,25,347^ For example, *NET1e* is highly expressed in breast cancer, and in situ overexpression of *NET1e* promotes cancer cell growth and drug resistance to the PI3K/mTOR inhibitor BEZ235 and the BCL2 inhibitor obatoclax.^24^ These observations indicate the appreciable potential of eRNAs for clinical utility in diagnostics and/or targeted therapies. In this review, we summarized the eRNAs that are differentially expressed across various cancers and outlined several approaches for targeting eRNAs. Nevertheless, there are still certain biological and technical hurdles to overcome for eRNA-targeted therapy.

One major concern regarding RNAi-based strategies is that siRNAs can potentially trigger unintended intracellular changes. For example, siRNAs may inadvertently degrade other transcripts owing to partial complementarity, leading to off-target effects,^348^ and high concentrations of siRNAs can disrupt the endogenous processing of microRNAs by overwhelming the RNAi machinery.^348^ The second bottleneck for eRNA therapy is the delivery challenge, encompassing issues such as instability of therapeutic agents, immune activation, poor cellular uptake, and the toxicity of delivery platforms.^311,312,349^ Once introduced into the bloodstream, naked siRNAs are rapidly degraded by nucleases and can simultaneously trigger an innate immune response.^348^ To address these issues, chemically modified ASOs have been developed to protect RNA from nuclease-mediated degradation and reduce immunogenicity.^350^ In addition, lipid nanoparticles (LNPs) and polymeric nanocarriers have been widely used to improve the stability of encapsulated RNA molecules.^351–353^ Co-delivery of biomolecules and cell-penetrating peptides has been shown to facilitate cellular uptake.^312^ However, whether these improvements can sufficiently target eRNAs in vivo remains unclear. Third, even though eRNAs exhibit cell-type-specific characteristics, the delivery of therapeutic agents in a tailored manner could further increase the targeting efficacy. To this end, one could consider conjugating the targeting biomolecules to cell-type-specific ligands to achieve better eRNA degradation efficiency. Advances in proteomic methods, such as ChIRP, RNA-agnostic profiling (RAP), and identification of direct RNA interacting proteins (iDRiP), may permit the identification of the critical protein partners of those clinically relevant eRNAs.^204,354,355^ Disrupting interactions between eRNAs and their key protein partners could also represent an effective strategy to interfere with eRNA-mediated oncogenic phenotypes.^356,357^

RIBOTACs offer a promising opportunity for targeted RNA degradation^358,359^ and have been widely applied in cancers,^315,360–362^ neurodegenerative diseases,^363,364^ and infectious diseases.^365,366^ Notably, DNA-encoded library (DEL) screening has been successfully repurposed to identify novel binders for PROTACs and RIBOTACs,^367–370^, accelerating the design of next-generation RIBOTACs capable of recruiting ribonucleases with specific characteristics, such as distinct subcellular localizations, tissue distributions, or substrate specificities. Notably, simply occupying the RNA target is often insufficient to disrupt RNA activity, especially when the functional site of the given RNA target has not been determined.^316,371,372^ Only a small fraction of targets bound by the RNA-binding module can be cleaved by the corresponding RIBOTACs in cells.^373^ Tong Y. et al. performed a global analysis to investigate the interplay between small molecule binding and RIBOTAC cleavage in live cells via an unbiased transcriptome-wide approach.^373^ Their study demonstrated that the cleaved targets generally form more stable structures and preferentially contain RNase L cleavage sites in close proximity to the small-molecule binding sites. In addition, the extent of cleavage is affected by the expression level of the target RNA.^373^ Furthermore, the selectivity of RIBOTACs is determined by the overall structure of the RNA target rather than just the local structural motifs recognized by the binder. The structural features adjacent to the RIBOTAC binding site may also alter the cleaving module.^374^ Taken together, these findings highlight the critical role of the RNA structure in the RIBOTAC-based RNA degradation strategy, which affects both its bioactivity and selectivity. However, RNAs often exist in dynamic structures.^375–377^ To this end, the same group employed a 15,000-member, natural-product-like small-molecule compound collection and a library of RNA 3D folds presented in a 3 × 3 internal loop library (ILL; 61,440,000 potential binding interactions probed) to define the structure–activity relationships between small molecules and their preferred RNA 3D folds.^360^ As a proof of concept, they showed that these biologically silent binders can be converted into potent RIBOTAC degraders that selectively downregulate disease-causing RNAs, for example, *JUN* and *MYC* mRNAs.^360^ This strategy further broadens the targeting scope of RIBOTACs, extending beyond functional sites to include structured RNA regions.^360^

Extensive ongoing efforts in the pharmaceutical industry have aimed to target disease-relevant RNAs.^378–380^ Advancements in RNA-targeting approaches, such as RIBOTAC, have opened new possibilities for investigating and modulating the function of specific eRNAs both in vitro and in vivo. Given the vital role of eRNAs in regulating gene transcription, particularly in human cancers, they hold unprecedented potential as therapeutic targets for cancer treatment, ultimately improving patient outcomes and expanding the landscape of RNA-based therapies.
